# Cytotoxic Chromosomal Targeting by CRISPR/Cas Systems Can Reshape Bacterial Genomes and Expel or Remodel Pathogenicity Islands

**DOI:** 10.1371/journal.pgen.1003454

**Published:** 2013-04-18

**Authors:** Reuben B. Vercoe, James T. Chang, Ron L. Dy, Corinda Taylor, Tamzin Gristwood, James S. Clulow, Corinna Richter, Rita Przybilski, Andrew R. Pitman, Peter C. Fineran

**Affiliations:** 1 Department of Microbiology and Immunology, University of Otago, Dunedin, New Zealand; 2 New Zealand Institute for Plant and Food Research, Christchurch, New Zealand; 3 Bio-Protection Research Centre, Lincoln University, Canterbury, New Zealand; Uppsala University, Sweden

## Abstract

In prokaryotes, clustered regularly interspaced short palindromic repeats (CRISPRs) and their associated (Cas) proteins constitute a defence system against bacteriophages and plasmids. CRISPR/Cas systems acquire short spacer sequences from foreign genetic elements and incorporate these into their CRISPR arrays, generating a memory of past invaders. Defence is provided by short non-coding RNAs that guide Cas proteins to cleave complementary nucleic acids. While most spacers are acquired from phages and plasmids, there are examples of spacers that match genes elsewhere in the host bacterial chromosome. In *Pectobacterium atrosepticum* the type I-F CRISPR/Cas system has acquired a self-complementary spacer that perfectly matches a protospacer target in a horizontally acquired island (HAI2) involved in plant pathogenicity. Given the paucity of experimental data about CRISPR/Cas–mediated chromosomal targeting, we examined this process by developing a tightly controlled system. Chromosomal targeting was highly toxic via targeting of DNA and resulted in growth inhibition and cellular filamentation. The toxic phenotype was avoided by mutations in the *cas* operon, the CRISPR repeats, the protospacer target, and protospacer-adjacent motif (PAM) beside the target. Indeed, the natural self-targeting spacer was non-toxic due to a single nucleotide mutation adjacent to the target in the PAM sequence. Furthermore, we show that chromosomal targeting can result in large-scale genomic alterations, including the remodelling or deletion of entire pre-existing pathogenicity islands. These features can be engineered for the targeted deletion of large regions of bacterial chromosomes. In conclusion, in DNA–targeting CRISPR/Cas systems, chromosomal interference is deleterious by causing DNA damage and providing a strong selective pressure for genome alterations, which may have consequences for bacterial evolution and pathogenicity.

## Author Summary

Bacteria have evolved mechanisms that provide protection from continual invasion by viruses and other foreign elements. Resistance systems, known as CRISPR/Cas, were recently discovered and equip bacteria and archaea with an “adaptive immune system.” This adaptive immunity provides a highly evolvable sequence-specific small RNA–based memory of past invasions by viruses and foreign genetic elements. There are many cases where these systems appear to target regions within the bacterial host's own genome (a possible autoimmunity), but the evolutionary rationale for this is unclear. Here, we demonstrate that CRISPR/Cas targeting of the host chromosome is highly toxic but that cells survive through mutations that alleviate the immune mechanism. We have used this phenotype to gain insight into how these systems function and show that large changes in the bacterial genome can occur. For example, targeting of a chromosomal pathogenicity island, important for virulence of the potato pathogen *Pectobacterium atrosepticum*, resulted in deletion of the island, which constituted ∼2% of the bacterial genome. These results have broad significance for the role of CRISPR/Cas systems and their impact on the evolution of bacterial genomes and virulence. In addition, this study demonstrates their potential as a tool for the targeted deletion of specific regions of bacterial chromosomes.

## Introduction

Prokaryotes are constantly challenged with foreign genetic elements such as bacteriophages (phages) and plasmids [1]. These interactions are frequent and important on a global scale. For example, of the estimated 10^31^ phages on earth, approximately 10^25^ participate in infections of bacteria every second [2] affecting biogeochemical cycles such as the carbon cycle [3]. The strong selective pressure has resulted in the evolution of numerous mechanisms of ‘innate immunity’ in bacteria [1], [4], such as abortive infection systems [5], and recent research has demonstrated that a prokaryotic ‘adaptive immune system’ exists. These ‘adaptive immune systems’, termed Clustered Regularly Interspaced Short Palindromic Repeats (CRISPRs), are a small RNA-based bacterial defence mechanism with some similarities to eukaryotic RNA interference and microRNAs (for reviews see [6]–[11]). Simply, CRISPRs are an important part of an ‘immune system’ with genetic memory against extrachromosomal agents such as plasmids and phages.

CRISPRs are found in ∼50% of sequenced bacteria and ∼85% of archaea [12] and are comprised of an AT-rich leader sequence of several hundred base pairs followed by short repeats interspersed with similar sized spacers of unique sequence. Spacers are derived from foreign nucleic acids and are important in the sequence-specific interference of phages and plasmids [9], [13]. Closely associated with CRISPRs are the *cas* genes (CRISPR associated) 14–16, which are necessary for resistance. CRISPR arrays and their associated *cas* genes are diverse, with CRISPR/Cas systems falling into three major types (I–III), which are divided into further subtypes [17], [18].

The characterised mechanism of CRISPR/Cas interference involves three phases; 1) resistance acquisition (spacer incorporation into the CRISPR array [11], [13]), 2) expression of *cas* genes and transcription and processing of the CRISPR arrays into small RNAs (crRNAs) [19]–[23] and 3) interference of either RNA [24]–[26] or DNA [27]–[29] at sites in the target element. Sequences in the invading element, from which spacers are derived and subsequently targeted, are termed protospacers. Adjacent to the protospacers, short motifs are present (termed CRISPR motifs or protospacer adjacent motifs (PAMs)), that are important for both incorporation [30]–[32] and targeting [33]–[35]. The resistance mechanism is mediated by the Cas proteins, many of which have been shown to interact as ribonucleoprotein complexes [19], [24], [26], [36]–[39].

CRISPR spacers that are homologous to database sequences are predicted to have targets in plasmids, phages and chromosomal genes. While CRISPR-interference of phages [13] and plasmids [28] has been proven, by comparison, the role of chromosomal targeting has received little attention. Initial analyses of CRISPR spacers showed that only ∼2% of spacers have identity to database sequences [9]. In lactic acid bacteria, of 104 spacers with 100% identity to databases, 73% matched with phage-related sequences, 5% with plasmids and 22% elsewhere within their own genome [40]. Another study showed that within archaea, 19% (of 58 matches) had identity elsewhere in the host chromosome [41]. This begs the question what role these chromosomal targeting spacers have. One early proposal was that CRISPRs might act as a gene regulation mechanism [7], but this has not yet been shown. A small number of studies indicate that chromosomal targeting can be detrimental [42]–[44] and a bioinformatic analysis of chromosomal targeting led to the suggestion that chromosomal targeting is a case of autoimmunity [45]. The authors proposed that spacer incorporation from chromosomal protospacers is lethal and, as such, they observed a correlation between mutations that were predicted to interfere with the hypothesised toxicity and chromosomally-derived spacers. These hypotheses have yet to be tested in wet-lab experiments.

In this study we have determined the effects of chromosomal targeting using both engineered and pre-existing spacers and tested the hypotheses proposed by Stern *et al* (2010). To investigate CRISPR/Cas-mediated chromosomal targeting we utilised the potato phytopathogen *Pectobacterium atrosepticum*, which contains a single type I-F (Ypest) CRISPR/Cas system composed of Cas1, a Cas2–Cas3 fusion, Csy1, Csy2, Csy3 and Cas6f (originally termed Csy4) and 3 CRISPR arrays with CRISPR-4 type repeats [46] and 28, 10 and 3 spacers [23], [47]. Previously, we demonstrated this CRISPR/Cas system is transcribed and the CRISPR arrays are processed into crRNAs by Cas6f [23]. *P. atrosepticum* contains one spacer with a perfect match to a chromosomal gene within a horizontally acquired island (HAI2). Here, we provide direct experimental evidence that the targeting of chromosomal genes by CRISPR/Cas systems is toxic and show the various mechanisms that enable avoidance of this autoimmunity, including the dramatic reshaping of pathogenicity islands within the bacterial genome. This suggests that CRISPR/Cas systems have played a greater role in bacterial genome evolution than previously appreciated. Furthermore, these experiments provide an insight into functional details of type I-F systems and show that CRISPR/Cas systems can be engineered to delete specific regions of bacterial genomes and thus, can provide a tool for genome engineering.

## Results

### Chromosome-targeting CRISPR/Cas systems are toxic

**Figure 1:**
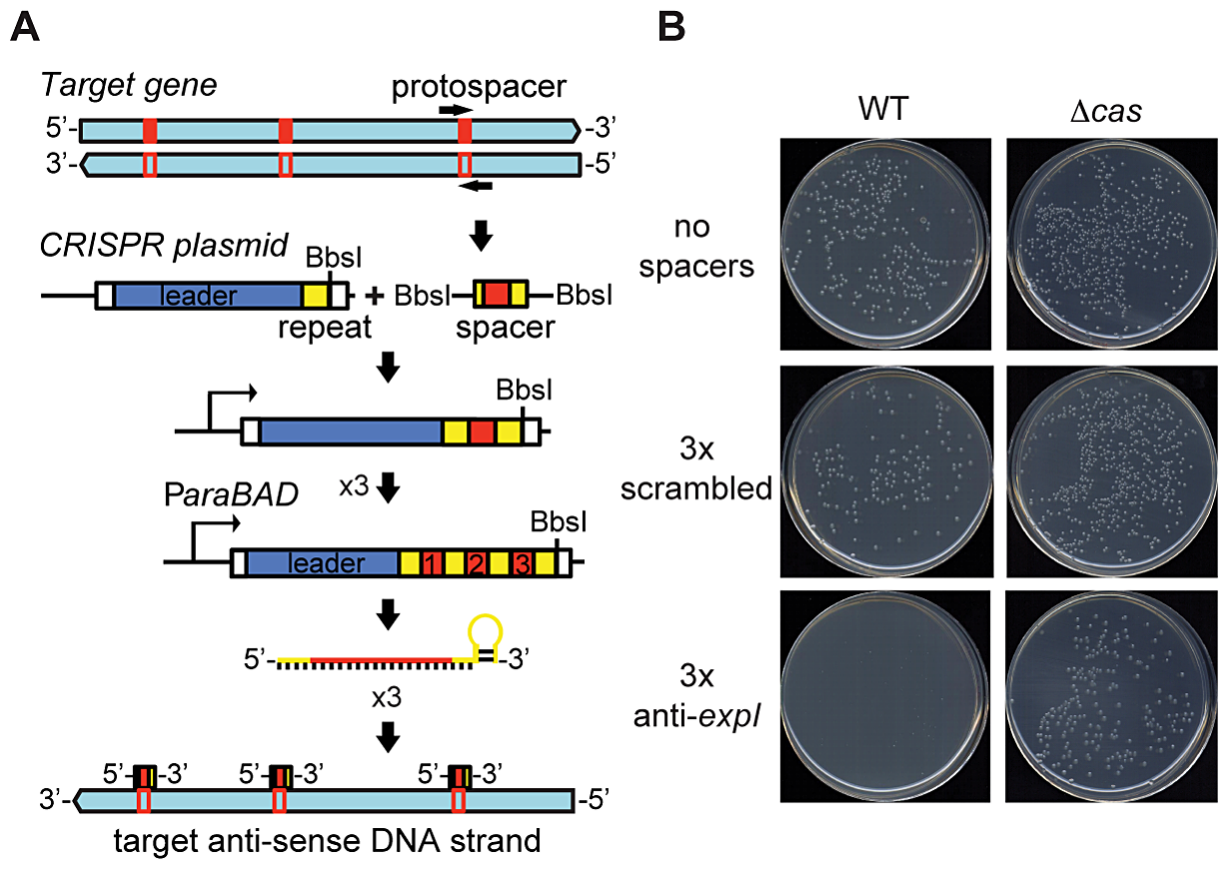
An engineered CRISPR plasmid with spacers targeting the chromosome displays Cas–dependent toxicity. (A) Strategy for chromosomal targeting (see Materials and Methods). Protospacers were PCR-amplified from target DNA with full or partial repeats and BbsI sites on the primers. PCR products were digested with BbsI and cloned into BbsI-digested plasmids containing a leader sequence and one repeat. This process was repeated to generate multiple spacer inserts. These arrays generate crRNAs that target the template DNA strand but not the mRNA. (B) Transformation plates of *P. atrosepticum* WT or a Δ*cas* mutant (PCF80) after growth for 36 h on LBA containing Ap. Plasmids transformed contained no spacers (pC1-780), 3 scrambled spacers (pS3-780) and 3 anti-*expI* spacers (pE3-780). Representatives are shown from experiments performed at least in triplicate.

To test the effect of chromosome-targeting by CRISPR/Cas systems, a strategy was developed to engineer CRISPR arrays (Materials and Methods) (Figure 1A). An array was engineered with the native type I-F CRISPR1 leader and three sense-orientation (i.e. cannot target mRNA) spacer-repeat units targeting the *expI* gene in *P. atrosepticum* (Figure 1A; spacer and PAMs indicated in Table S1). The *expI* gene encodes the *N*-acyl homoserine lactone synthase, which produces quorum sensing signals [48]. When the *expI-*targeting plasmid was transformed into *P. atrosepticum*, the efficiency was similar (Figure S1) but transformants were almost undetectable compared with control plasmids containing either no spacers, or three spacers that do not match chromosomal targets (a scrambled control) (Figure 1B). To determine if the toxicity was Cas-dependent, the effect of deleting the *cas* operon was assessed. The toxic effect of the chromosomal *expI*-targeting plasmid was abolished by deletion of the *cas* operon (Figure 1B). Therefore, CRISPR/Cas systems with specific spacers that target the chromosome are detrimental to bacterial growth.

### Chromosomal targeting causes growth inhibition

**Figure 2:**
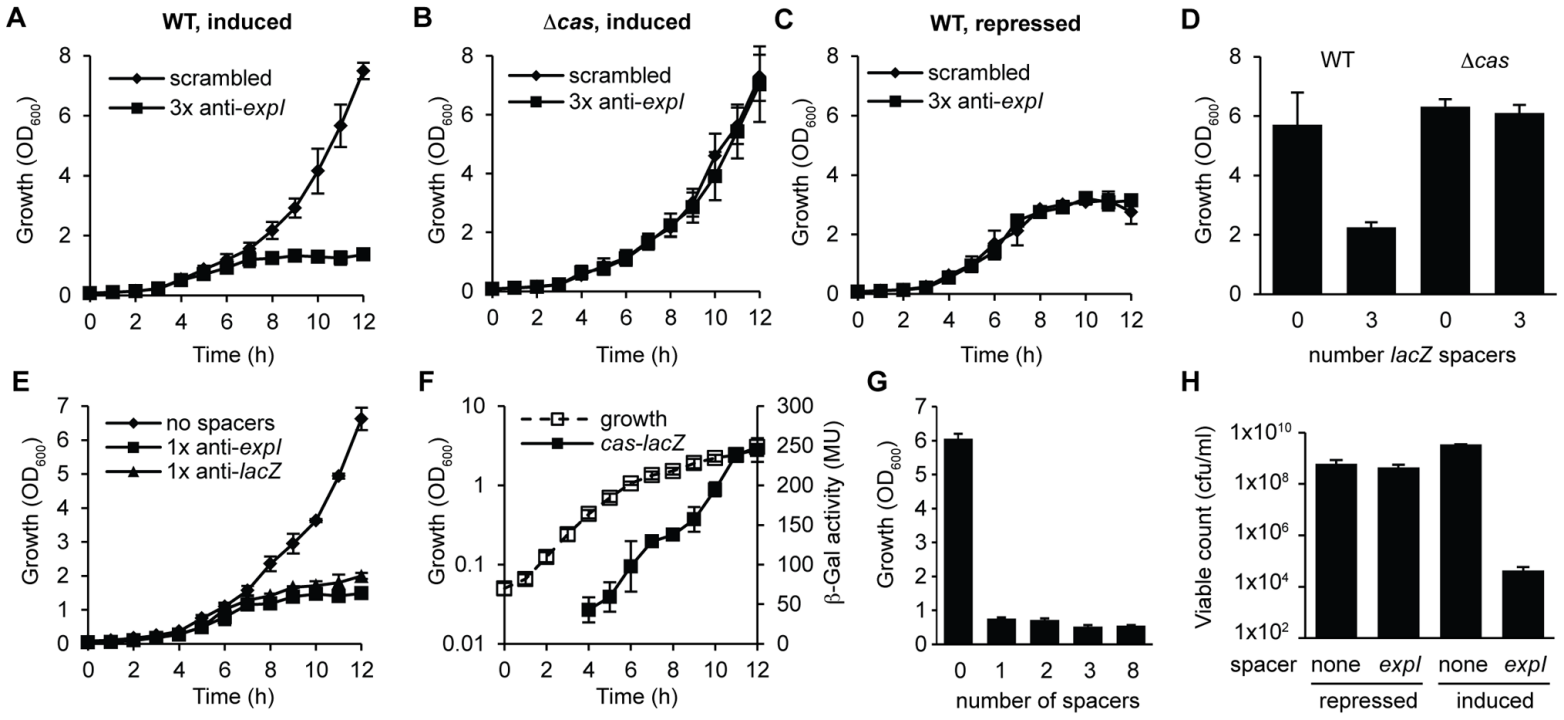
Controlled expression of single anti-***expI*** **or anti-** ***lacZ*** **spacers causes** ***cas*** **–dependent growth inhibition and a reduction in viable count.** Plasmids containing either 3 scrambled spacers (pS3-16) or 3 spacers targeting *expI* (pE3-16) were induced in either (A) WT or (B) Δ*cas* (PCF80) strains or (C) repressed in the WT. (D) Plasmids containing either no spacers (pC1-16) or 3 spacers targeting *lacZ* (pL3-16) were induced in either WT or Δ*cas* strains and measured after 12 h. (E) Plasmids containing no spacers (pC1-16) or one spacer targeting *expI* (pE1-16) or *lacZ* (pL1-16) were induced in the WT. (F) Expression of the *cas* operon measured from a chromosomal transcriptional/translational *lacZ* fusion (PCF79). (G) Plasmids containing no spacers (pC1-16) or one, two, three or eight identical spacers (pTraG1-16, pTraG2-16, pTraG3-16 and pTraG8-16) display similar toxicity. (H) Viable count (cfu/ml) after 12 h with either repression or induction of plasmids encoding no spacers (none) or 1× anti-*expI* (*expI*). Limit of detection was 1×10^2^. Data shown are the mean ± the SD of three experiments.

The engineered CRISPR array included 780 bp of sequence 5′ of the first proximal repeat. Attempts to repress expression from these pBAD30-derived plasmids did not abolish toxicity (Figure S2A), supporting our previous assignment of the CRISPR1 promoter within ∼180 bp of leader [23]. To develop tightly-controlled CRISPRs, a plasmid truncation series was produced with 780, 180, 52 and 16 bp 5′ of the first repeat. Only plasmids with 16 bp were controllable, which led to the identification of a putative CRISPR1 promoter within 52 bp of the leader (Figure S2). In the WT background, controlled induction of the *expI*-targeting plasmid resulted in a cessation of growth (a plateau in OD_600_) compared with the scrambled control (Figure 2A). Furthermore, no growth inhibition occurred in the *cas* mutant, or in the WT when grown under repressed conditions (Figure 2B and 2C). A *lacZ*-targeting array was constructed and was also toxic in the WT but not the Δ*cas* mutant (Figure 2D). Note that *P. atrosepticum* utilises arabinose and grows to a higher OD_600_ when compared with the repressed (glucose-grown) controls.

We hypothesized that a single spacer would be sufficient for targeting, since the 3× anti-*lacZ* plasmid contained only one spacer complementary to a protospacer with a consensus PAM (protospacers contained 5′-protospacer-AC-3′ and 5′-protospacer-GT-3′ PAMs and the consensus 5′-protospacer-GG-3′ PAM [33], [49] (Table S1; the protospacer is defined as the target strand complementary to the crRNA and the PAM is denoted 5′-3′ on this strand [8]). In agreement, expression of CRISPRs containing only one spacer against either *expI* or *lacZ* inhibited bacterial growth (Figure 2E). Controls using either repressed conditions or in the Δ*cas* strain always demonstrated no effect of the chromosomal-targeting plasmids, as shown in Figure 2A–2D, and are therefore not shown for clarity here and in later figures. The growth inhibition, as measured by OD_600_, was detected around mid-exponential phase (6–7 h) for both targets, which corresponds with the increased expression of a native *cas* operon measured using a chromosomal *cas-lacZ* transcriptional/translational reporter (Figure 2F). With plasmids containing one, two, three or eight identical chromosomal targeting spacers there was no apparent additional effect (Figure 2G). The reduced OD_600_ resulting from a single spacer targeting *expI*, was reflected in a ∼10^5^ reduction in viable count (cfu/ml) (Figure 2H). The viable counts were assessed on media that repressed crRNA synthesis, indicating that most cells could not readily recover following chromosomal targeting, but that a subpopulation survived. The initial inoculum in these experiments was ∼10^7^ cfu/ml and the final viable count following targeting was ∼10^4^ cfu/ml compared with ∼10^9^ cfu/ml for the negative controls. Together, these experiments demonstrate that a single spacer that targets the chromosome causes *cas*-dependent toxicity and a reduction in viable count.

### Chromosomal targeting causes cellular elongation

**Figure 3:**
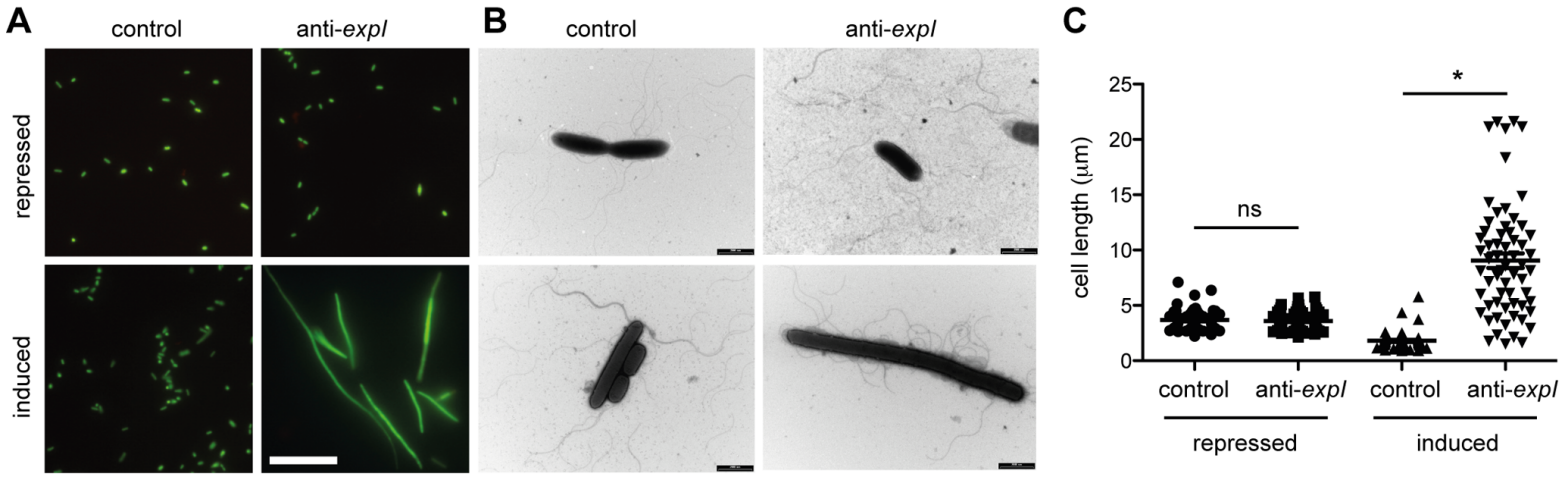
Chromosomal targeting results in cell elongation indicative of DNA damage. Plasmids containing no spacers (control; pC1-16) or one spacer targeting *expI* (anti-*expI*; pE1-16) were repressed or induced in the WT and then visualised by (A) LIVE/DEAD staining and fluorescence microscopy (white scale bar; 20 µm; all images to scale) or (B) transmission electron microscopy (TEM) (black scale bars; 2 µm) (C) Quantification of cell lengths of 60 cells from each treatment as assessed by TEM. ns, not significant; *, p-value of <0.0001 when assessed by unpaired two-tailed t-test (PRISM).

The toxicity following chromosomal targeting prompted an examination of morphological changes to the cells. LIVE/DEAD staining and fluorescence microscopy following 2 h of expression of a single spacer targeting *expI* led to the detection of elongated/filamentous cells (Figure 3A). Interestingly, most filamentous cells were stained with SYTO9, but not propidium iodide (PI), indicating that, of the filamentous cells detected, they were still viable and maintaining membrane integrity (Figure 3A). In all conditions including controls, a few cells were stained with PI, suggesting loss of viability in a subpopulation. Following chromosomal targeting, cells were also imaged by transmission electron microscopy (TEM) (Figure 3B). Targeting of *expI* significantly increased the mean cell length to ∼10 µm compared with ∼2 µm with the non-targeting control, but some cells were over 20 µm (Figure 3C). Therefore, chromosomal targeting caused a dramatic reduction in viable count and had a bacteriostatic effect on some cells, resulting in elongation. This cell elongation is likely to explain the slight increase in OD_600_ measurements prior to the plateau in growth (e.g. see Figure 2A). Cellular filamentation in *E. coli* is indicative of DNA damage and induction of the SOS response [50], which is consistent with a DNA target for type I-F systems.

### The effect of protospacer and PAM mutations on toxicity

**Figure 4:**
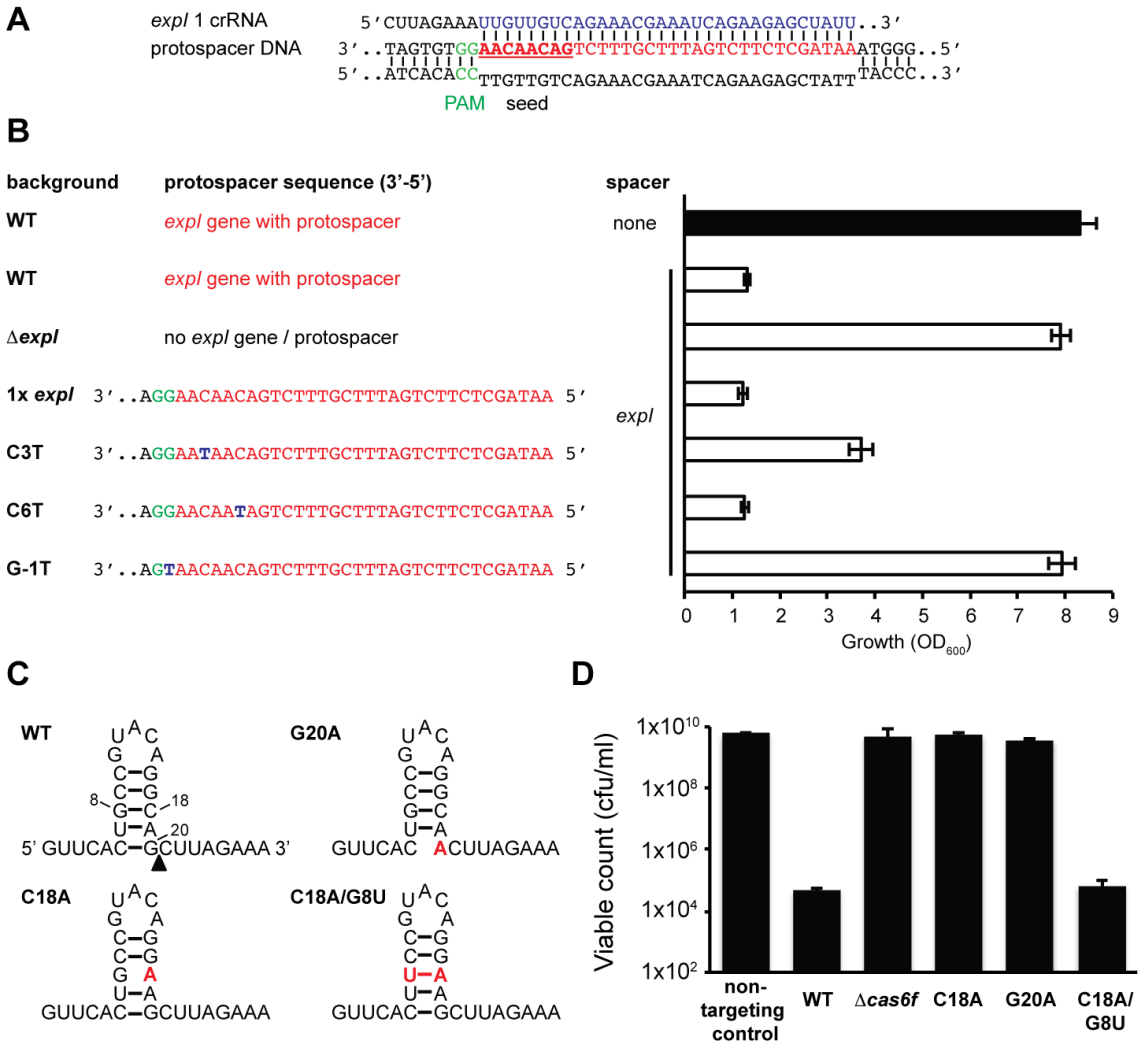
Protospacer, PAM, and repeat mutants can escape toxicity. (A) Predicted pairing between an *expI* crRNA and the *expI* protospacer (PAM is green, spacer is blue, protospacer is red and seed sequence is bold and underlined). (B) Left; protospacer sequences in WT, Δ*expI* (PCF81), Δ*expI* with a single WT (RBV01), C3T (RBV04), C6T (RBV03) or G-1T (PAM) (RBV02) *expI* protospacer. Right; toxicity assays of a single anti-*expI* spacer (*expI*; pE1-16; white bars) or a plasmid with no spacers (none; pC1-16; black bar) when expressed in the backgrounds shown on the left. (C) Predicted folds and position of mutations for WT, G20A, C18A and C18A/G8U single CRISPR repeats. The black triangle represents the site of cleavage by Cas6f. (D) Toxicity assays of plasmids expressing a single anti-*expI* spacer flanked by either WT (pE1-16), G20A (pE1-16 G20A), C18A (pE1-16 C18A) and C18A/G8U repeats (pE1-16 C18A/G8U) in WT *P. atrosepticum*.

The detrimental effect of chromosomal targeting indicated that multiple mutational routes should lead to CRISPR/Cas avoidance. Such mutations have been predicted to include the *cas* genes, protospacer, PAM, and the repeats/crRNA processing [45]. We showed that mutation of the *cas* operon abrogates toxicity (Figure 1 and Figure 2). To test protospacer mutations, first the entire *expI* target gene (including the protospacer) was deleted from the chromosome. In this Δ*expI* strain, the toxicity induced by a single *expI* spacer was abolished (Figure 4A and 4B). Re-introduction of a single *expI* protospacer with an optimal 5′-protospacer-GG-3′ PAM restored toxicity, confirming that the protospacer enabled targeting (Figure 4A and 4B). Previously, a ‘seed’ sequence of 8 nt in the spacer, adjacent to the 5′ handle, has been shown to be important for the initial binding of the crRNA to the target in the type I-E system [35]. In the type I-F system, the seed is less well defined, but a short ssDNA substrate of nucleotides 1–8 bound the Csy complex with highest affinity [38]. To test the role of the seed sequence, two seed sequence protospacer mutations were generated in the chromosome and tested for interference. A C3T mutation resulted in partial avoidance of targeting when measured by OD_600_ (Figure 4B) or by viable count (an 100-fold reduction in viable count for the C3T PAM mutant compared with 10^4^ to 10^5^-fold reductions for WT protospacers). Next, we tested a C6T mutation, which did not affect targeting/toxicity, demonstrating that a level of mismatch is tolerated in the type I-F seed sequence (Figure 4B). This result mirrors the tolerance observed at the identical position in the *E. coli* type I-E system [35] and is consistent with the existence of a discontinuous seed region in type I-F systems.

PAM sequences are required for interference of plasmids and phages [34], [35]. Evidence that the PAM is required in the type I-F systems was provided by the fact that re-introduction of an *expI* protospacer containing a single PAM nucleotide substitution mutation protected this strain from chromosomal targeting (Figure 4B). This shows that a single mutation in the type I-F PAM is sufficient to escape targeting. Cady et al. recently identified G-1A PAM (e.g. 5′-protospacer-AG-3′) phage escape mutants in the background of other protospacer mismatches [51]. Therefore, deletion or mutation of the protospacer target and PAM mutations can alleviate targeting and growth inhibition.

### CRISPR repeat or *cas6f* mutations abolish toxicity

Mutations in CRISPR repeats can inhibit pre-crRNA processing and crRNA generation and hence interfere with chromosomal targeting. Mutation(s) were introduced in both repeats flanking a single *expI* spacer (Figure 4C). Firstly, a G20A mutation, that was previously shown to abrogate Cas6f (Csy4)-dependent endonucleolytic cleavage [22], abolished targeting (Figure 4D). Next, we predicted that a C18A mutation would destabilise the crRNA stem-loop secondary structure, abolish processing and chromosomal targeting. Indeed, the C18A mutant was non-toxic (Figure 4D). By introducing a compensatory mutation (C18A/G8U), toxicity was restored, demonstrating that the repeat RNA stem-loop secondary structure was important but the sequence was not essential. Interestingly, a recent report showed the C18A/G8U mutation in *P. aeruginosa* subtly affected RNA binding and cleavage by Cas6f [52]. Apparently, this 2-fold reduced cleavage is sufficient for crRNA generation and toxicity (Figure 4D). To test the involvement of Cas6f in crRNA generation, and hence toxicity, targeting in a Δ*cas6f* mutant was assessed. We previously showed that deletion of *cas6f* abolished crRNA generation in *P. atrosepticum* [23]. As expected, chromosomal targeting was absent in the Δ*cas6f* strain (Figure 4D). In summary, particular mutations of repeats or the endoribonuclease provides protection from CRISPR/Cas-mediated chromosomal targeting.

### A single nucleotide PAM mutation enables escape from native CRISPR/Cas targeting

**Figure 5:**
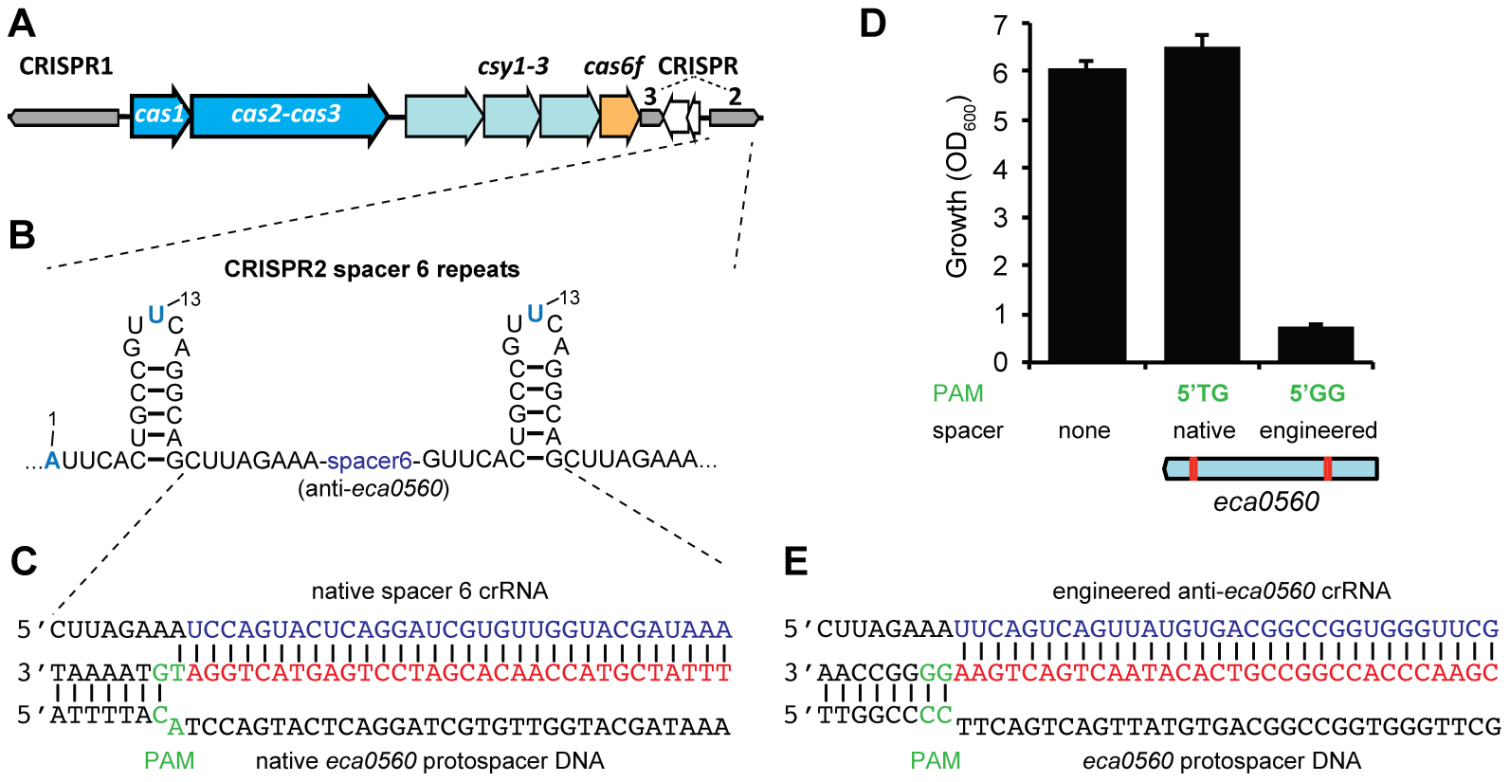
A single nucleotide PAM mutation enables escape from native CRISPR/Cas targeting. (A) *P. atrosepticum* CRISPR/Cas genomic organisation. The *cas1* and the *cas2*-*cas3* hybrid genes are shown in blue, *csy1*-*3* in pale blue, *cas6f* in orange and the CRISPRs as grey arrows in the direction of transcription. CRISPR2 and 3 are separated by a toxin/antitoxin system (white arrows). (B) Spacer 6 of CRISPR2 (from leader) contains deviations from the repeat consensus (shown in blue) and (C) has a 100% match to a protospacer (red) within *eca0560* in HAI2 in the *P. atrosepticum* genome. The protospacer matching spacer 6 contains a non-consensus 5′-protospacer-TG-3′ PAM (green). (D) Toxicity assays in the WT with plasmids containing consensus repeats and either no spacer (pC1-16), a single native spacer 6 (5′-protospacer-TG-3′ PAM; pTraGS6-16) or an engineered spacer (5′-protospacer-GG-3′ PAM; pTraG1-16) that targets *eca0560*. The protospacer locations in *eca0560* of the native (pTraGS6-16) and engineered (pTraG1-16) spacers are shown below in red. (E) Sequence and pairing of the engineered anti-*eca0560* spacer (in pTraG1-16) with the consensus 5′-protospacer-GG-3′ PAM (green).

Spacer 6 in CRISPR2 has a 100% match to *eca0560* in the *P. atrosepticum* genome within an ∼100 kb horizontally acquired island named HAI2 (Figure 5A–5C) [23]. The function of ECA0560, a TraG-family protein, is unknown but it is highly conserved in Integrative Conjugative Elements (ICE) [53], such as HAI2, and is predicted to be involved in their mobility. HAI2 contains the *cfa* gene cluster involved in the biosynthesis of coronafacic acid, a polyketide phytotoxin important for plant pathogenicity in potato [54]. Since we demonstrated chromosomal targeting is toxic, we hypothesised that this spacer is non-toxic due to mutations that might interfere with the targeting mechanism. Clearly, the *cas* genes are functional, given our engineered assays (see Figure 2). However, the repeats adjacent to spacer 6 contained mutations (Figure 5B) and the PAM was not the type I-F consensus (Figure 5C) [33].

Firstly, we examined if the G1A and A13U repeat mutations affected targeting. The repeat mutations were “repaired” by cloning spacer 6 between two WT CRISPR1 consensus repeats. When expression of spacer 6 with consensus repeats was induced in the WT, no toxicity occurred (Figure 5C and 5D). This suggested that the repeat mutations were not the cause of tolerance to this spacer. Indeed, in an *in vitro* assay, Cas6f cleaved pre-crRNA transcripts covering repeats either side of either spacer 2 (control) or spacer 6 (Figure S3), indicating that these mutations do not inhibit endonucleolytic processing to yield spacer 6 crRNAs. Together, these results show that the inability of this spacer to target the chromosome was not due to repeat mutations.

Next, the role of the PAM was assessed. The protospacer had a non-consensus type I-F PAM of 5′-protospacer-TG-3′ (Figure 5C) compared with the consensus of 5′-protospacer-GG-3′ [33] (Figure 5D). To test if the 5′-protospacer-TG-3′ PAM accounted for the lack of targeting, a single spacer was engineered against a 5′-protospacer-GG-3′ in *eca0560* (Figure 5E). This engineered spacer caused a toxic effect on *P. atrosepticum* when compared with the 5′-protospacer-TG-3′ non-consensus PAM and a no spacer control (Figure 5D). This result is in agreement with our single nt mutation introduced in the PAM of the *expI*-targeting spacer, which abolished targeting (Figure 4) and with two other recent studies [49], [51]. In summary, these results show that targeting HAI2 is toxic to *P. atrosepticum*, but that a non-optimal PAM sequence present in the protospacer of CRISPR2 spacer 6 has allowed evasion from interference.

### CRISPR/Cas–mediated chromosomal targeting causes rapid genome evolution

Our demonstration that CRISPR/Cas systems can target host genomes and cause profound growth inhibitory effects that are avoided by a range of mutations led us to ask whether chromosomal targeting can drive genome evolution due to spontaneous target site deletion. Specifically, we tested if targeting of the HAI2 pathogenicity island could result in complete loss, or internal deletions within the island. We used a strain with a Km^R^ cassette in *eca0573*, a gene within HAI2, which provided a marker to screen for island loss. Expression of the engineered crRNA targeting *eca0560* (in HAI2) led to growth inhibition (e.g. Figure 5D), but when cultures were left for 36 h, suppressor mutants arose. Twenty isolates from 600 survivor colonies (12 independent experiments) were sensitive to kanamycin, suggesting that loss of the *eca0573* Km^R^ marker had occurred. In control experiments with a non-targeting plasmid (pBAD30) all 600 isolates retained kanamycin resistance. We assumed that the loss of Km^R^ was due to specific targeting of HAI2. However, it was possible that general DNA damage and stress, caused by chromosomal interference, promoted the loss of the kanamycin marker. When we targeted *expI* (*eca0105*) elsewhere in the genome, instead of *eca0560*, none of the 600 survivors had lost the kanamycin resistance marker in *eca0573*, supporting that avoidance of specific targeting occurs by protospacer deletion and flanking DNA sequences.

**Figure 6:**
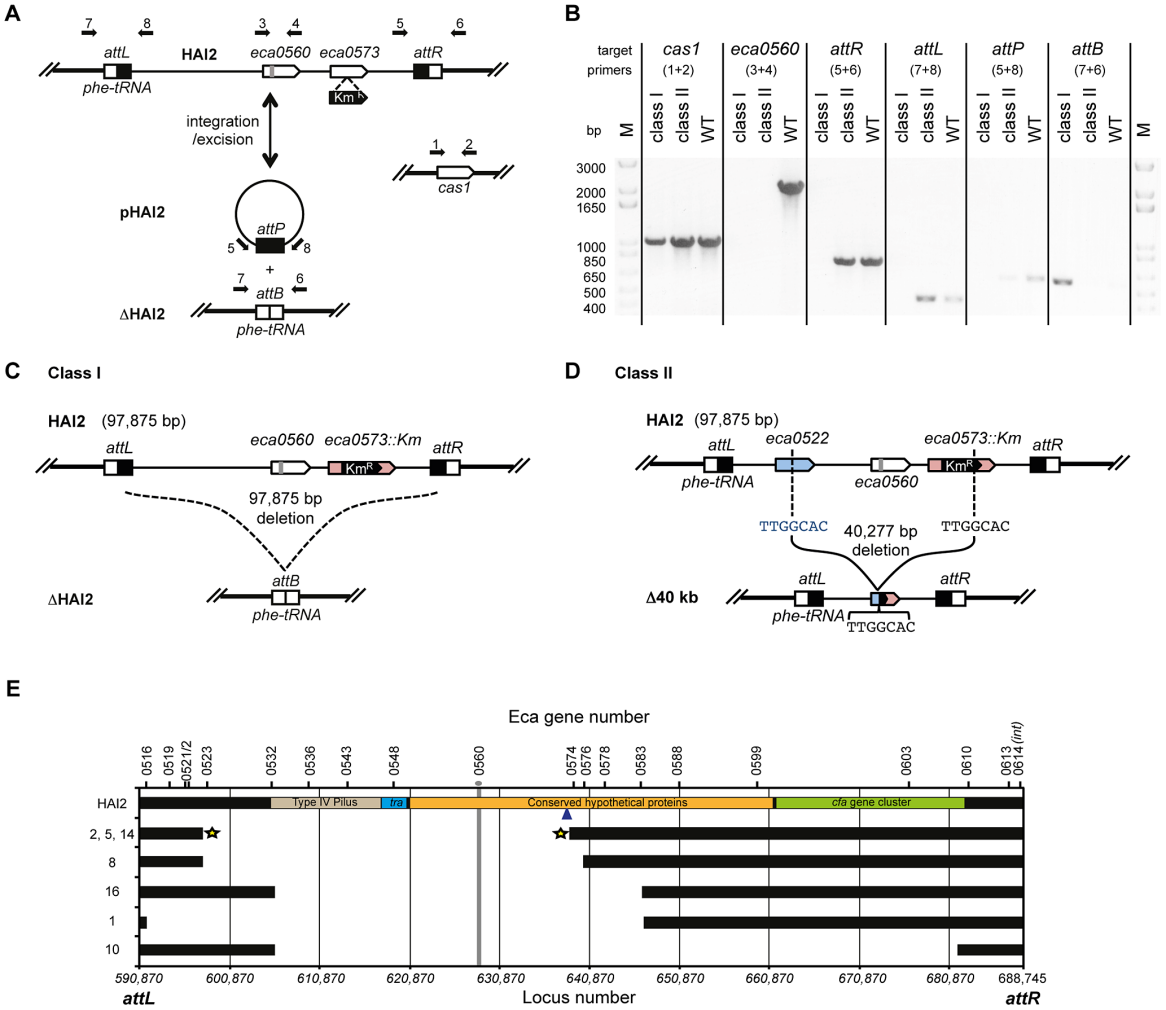
CRISPR/Cas–mediated chromosomal targeting causes rapid genome evolution. (A) Schematic of HAI2 inserted in the *P. atrosepticum* genome. The *attL* and *attR* sites are indicated by black and white boxes respectively, *eca0560* and *eca0573* are shown as white arrows and the protospacer in *eca0560* is indicated in grey and the Km^R^ marker in *eca0573* is depicted in black. Excision of HAI2 results in the circularised form, pHAI2, which contains the *attP* site and results in the generation of the *attB* site located within the phe-tRNA gene in the genome. In the absence of the circularised pHAI2 form, the strain is designated **Δ**HAI2. Primers used for strain confirmation in (B) are shown as black arrows and the *cas1* gene was used as a positive control. (B) PCR results for representative class I and class II mutants and the WT. PCR was performed for *cas1*, *eca0560*, *attR*, *attL*, *attP* and *attB* and with primers shown in part (A). Schematic representations of (C) the class I mutants (designated **Δ**HAI2) that have precisely lost the 97,875 bp island and (D) the class II mutants 2, 5 and 14 containing a 40,227 bp deletion between TTGGCAC sequences in both *eca0522* and internal to the Km^R^ insertion in *eca0573*. (E) Scale genetic map of the 7 classified HAI2 class II mutants defining the deleted regions. Black bars indicate the presence of the gene as specified. The gray vertical line represents the CRISPR-targeted *eca0560* gene. The star represents three accurately sequenced junctions. The blue arrow depicts the site of Km insertion within the chromosome. Class II mutants are numbered as in Figure S4 and their PCR profiles are shown in Figure S5.

Next, we examined how the mutants had avoided CRISPR-targeting. HAI2 inserts into the *P. atrosepticum* genome by site-specific recombination between the *attP* (plasmid) site in circularised pHAI2 and the *attB* (bacteria) site in the phenylalanine tRNA gene in the chromosome. The resulting linearised form of HAI2 is flanked by *attL* (left) and *attR* (right) sites, which are composites of the original *attP* and *attB* sites. By using combinations of primers that assess the presence of *attB*, *attP*, *attL*, *attR*, *eca0560* (target gene) and *cas1* (control), the presence or absence of the entire HAI2 or the target gene was determined (Figure 6A). Two major classes of mutants were present within the 20 survivors (see Figure 6B–D). There were 13 class I mutants that had lost the entire pathogenicity island (**Δ**HAI2) (Figure S4A), whereas 7 class II mutants were identified, which had lost kanamycin resistance and the target gene *eca0560*, but retained *attL* and *attR*, suggesting an internal HAI2 mutation (Figure S4B). The PCR result for a representative from both classes is shown in Figure 6B. The *attB* PCR product from multiple **Δ**HAI2 strains was sequenced (Figure S4C), demonstrating that HAI2 had been lost by a precise excision event, but that the excised pHAI2 form (i.e. *attP*) had been eliminated from these strains (Figure 6B and 6C). In WT strains there is a low frequency of HAI2 excision (∼10^−6^) [53], which can be detected as faint *attB* and *attP* PCR products. However, when HAI2 is lost entirely, *attB* is strongly amplified and no *attP* product is detected, testament to the loss of this pathogenicity island.

We also mapped the deletions in the class II mutants that retained a portion of the island (*attL* and *attR*) but lacked *eca0560* and kanamycin resistance. By using extensive combinations of primer pairs for genes in different parts of HAI2 (Figure S5), we could determine which regions were still present and which were absent (Figure 6E). These analyses demonstrated 5 different deletions amongst these 7 mutants. Mutant 10 has the largest deletion and has lost up to 78 genes including the coronafacic acid (*cfa*) cluster. Surprisingly, all 7 mutants retain the ability to generate excised derivatives of pHAI2, as detected in an *attP* PCR, despite lacking many genes including several belonging to the syntenic core [55] (Figure S4B). All mutants also lack the type IV pilus genes indicating that they are unlikely to be self-transmissible [56]. To map the deleted region precisely, primers on either end of the predicted deletion sites were used in PCR and the resulting products sequenced. In this manner, mutants 2, 5 and 14 had the exact deletion junction sequenced. These mutants all contained a deletion from 596727 to 637003 within the published *P. atrosepticum* SCRI1043 sequence [54], which corresponded to deletion within *eca0522* to a site within *eca0573*. Part of the Km^R^ insertion (289 bp) was still present, which included a 5′-TTGGCAC-3′ heptanucleotide sequence at the site of deletion that might have facilitated the recombination/DNA repair with *eca0522* following crRNA interference of *eca0560* (Figure 6D and 6E). The resulting strains have deleted 40,277 bp, 51 entire genes and 2 partial genes.

The question remained what the impact would be if non-mobile regions of the genome were targeted. To test targeting of non-mobile regions, a strain with a single WT *expI* protospacer immediately adjacent to a *cat* cassette was used (RBV01). This strain was targeted with the complementary *expI* crRNA (pE1-16) and survivors were screened for loss of the linked chloramphenicol resistance gene. Of 624 survivors screened, 23% were Cm^S^, indicating that deletion of *cat* and other genomic regions does occur to enable the evasion of chromosomal targeting. The higher frequency of Cm^S^ (23%) compared with Km^S^ (3%) survivors in the *expI* and *eca0560* targeting experiments might be a result of the linkage distance between the markers and the target site (immediately adjacent for *expI* and ∼10 kb for *eca0560*). To test a markerless system, the *lacZ* gene (*eca1490*) was targeted with a single crRNA (pL1-16). Ten survivors that were white on X-gal plates were analysed by PCR in more detail (Figure S6). All mutants retained *cas1* (*eca3679*; control), yet had lost the *lacZ* gene and >50 kb of chromosomal sequence 5′ of *lacZ* including *lacY* (*eca1489*), two large non-ribosomal peptide synthetases (NRPS) (*eca1488* and *eca1487*) and genes *eca1486-eca1482* (Figure S6). The extent of these deletions was not characterized, but it is apparent that targeting other regions of the chromosome can also result in large changes in genomic content. The role of this NRPS region is unknown, but has similarities to others that produce secondary metabolites required for pathogenicity.

In summary, the toxic effect elicited by CRISPR/Cas-mediated chromosomal targeting provides a strong selective pressure for the loss of the protospacer target. This can result in large-scale genomic changes which include precise excision and loss of pathogenicity islands, their modification, or deletion of other regions of the chromosome. Therefore, CRISPR systems are likely to have played an additional and greater role in the evolution of bacterial genomes than previously thought.

## Discussion

In this study we set out to examine the effects of chromosomal targeting by CRISPR/Cas systems. A bioinformatic approach to this question had previously led to the suggestion that incorporation of spacers that match the chromosome is accidental, resulting in a detrimental interference effect and the selection for mutants that have inactivated targeting [45]. We showed directly that CRISPR/Cas targeting of the chromosome is toxic and that mutations that disrupt the CRISPR/Cas mechanism enable cell survival. Importantly, we demonstrate that the negative fitness cost associated with chromosomal targeting can provide a strong selective advantage for strains lacking the target DNA. This selective pressure can result in large-scale genomic changes, including the deletion and remodelling of pathogenicity islands and hence, CRISPR/Cas can influence bacterial genome evolution that may lead to changes in virulence. Furthermore, this strong selection provides a tool for the deletion of targeted regions of bacterial genomes.

A novel method was developed to generate tightly-controlled crRNA expression vectors for cloning of multiple spacer sequences (Figure 1). By using these vectors to express crRNAs against three independent non-essential host genes in *P. atrosepticum*, we provide direct experimental evidence that chromosomal targeting by CRISPR/Cas systems is highly detrimental to bacterial growth and viability. Toxicity is a CRISPR/Cas-dependent process since deletion of the *cas* operon, the absence of the targeting crRNA or the presence of non-targeting (scrambled) spacers all led to a non-toxic effect (Figure 1, Figure 2, Figure 3).

Expression of chromosome-targeting crRNAs resulted in a non-reversible ∼10^5^ reduction in viable counts (Figure 2H), but some cells continued to grow and elongate (Figure 3). The filamentation phenotype was reminiscent of defects caused during the SOS response triggered by DNA damage [50], in accordance with DNA as the target of type I-F systems. Although there is no direct evidence, DNA is the likely target for type I-F CRISPR/Cas systems. For example, *in vitro*, the *P. aeruginosa* type I-F ribonucleoprotein Csy complex bound DNA that was complementary to the crRNA spacer [38]. However, in another study it was proposed that RNA is targeted [57], but recent work by the same group demonstrated plasmid and phage interference [51], consistent with a DNA target. The spacers in our study were designed sense to the mRNA of non-essential genes and therefore, could only base pair with the template strand of DNA. Therefore, the toxic and cell elongation phenotypes observed upon chromosomal targeting provide further evidence that DNA is targeted by type I-F systems.

Our molecular and genetic analyses have directly tested the bioinformatic hypotheses put forth by Stern et al. (2010) that chromosomal targeting is detrimental rather than regulatory. This model predicts that chromosomal targeting results from the accidental incorporation of host DNA as spacers and only cells that acquire mutations that deactivate targeting will survive. We unambiguously show that mutation of the *cas* genes, PAM, protospacer and repeats alleviate the severe fitness cost associated with self-targeting. However, it is tempting to speculate that there could be evolutionary advantages of self-targeting. In particular environments, chromosomal targeting might result in enhanced adaptive plasticity by enabling a subpopulation of cells to remove or remodel genomic regions associated with reduced fitness.

Our experiments have also provided insight into mechanistic details of the type I-F systems. For example, we demonstrated a role for the protospacer, specifically the seed sequence, and the PAM in interference and that single nucleotide mutations in the seed sequence can have varying effects on targeting by the type I-F system (Figure 4). Interference is typically viewed as an all-or-none phenomenon, and in the type I-E system, a single C3A protospacer mutation enabled phage M13 infection [35]. We showed a C3T mutation led to partial interference, which either highlights differences between type I-E and I-F systems, or might indicate that intermediate effects exist, depending on the mismatched nucleotide or the greater sequence context. An intermediate phage resistance was reported with multiple protospacer mutations in the type I-F system of *P. aeruginosa* [51]. The role of the repeats and the Cas6f endoribonuclease on chromosomal targeting was also determined. Mutation of *cas6f*, a CRISPR repeat C20A mutation, known to abolish processing [22], or a C18A repeat mutation all led to protection from self-targeting. In contrast, single repeat mutations G1A and A13U or a C18A/G8U double mutant were all functional, confirming a degree of flexibility of Cas6f proteins for their substrate RNA [52]. These results highlight that a more detailed understanding of specific endoribonucleases is required before assuming that defective processing will result from repeat mutations [45].

Bioinformatic studies have provided some insight into the effects of chromosomal targeting. Some *E. coli* and *Salmonella* strains contain type I-F arrays, but lack the type I-F *cas* genes. In these cases, spacers with homology to the type I-F genes are present in the orphan arrays and two non-mutually exclusive theories have been suggested. One theory is that self-*cas* targeting and subsequent deletion of the *cas* genes occurred [58], which is supported by our data and might help explain why roughly half of bacteria lack CRISPR/Cas. Alternatively, these orphan arrays might target and inhibit acquisition of plasmids encoding type I-F *cas* genes [59], [60]. An anti-CRISPR/Cas role was also proposed in archaea, where multiple spacers matched ORFs in other genomes and a subset had similarity to *cas* genes from other CRISPR/Cas systems [61]. However, it is also feasible that some of these ORFs were previously chromosomal and have been eliminated by CRISPR/Cas genomic targeting.

A small number of experimental studies have suggested chromosomal targeting causes a toxic effect. In *Pelobacter carbinolicus*, one spacer in the type I-E system matches the *hisS* gene [42], which contains the consensus 5′-protospacer-CTT-3′ PAM. Transformation with a plasmid containing an anti-*hisS* spacer into a *G. sulfurreducens* strain, which contained the *P. carbinolicus hisS* gene, was reduced compared with a vector control [42]. Also in a type I-E system, expression of engineered crRNAs targeting a lambda prophage in *E. coli* led to an ∼2 log reduction in viable count compared with control spacers [43] and has been used as a positive selection for CRISPR-inactive mutants [62]. In *Sulfolobus solfataricus*, transfection with arrays targeting the host β-galactosidase gene caused a growth reduction and resulted in elimination of the artificial arrays by recombination with the native CRISPRs [44]. In these studies, the data indicates that targeting host (or integrated; e.g. *hisS* and lambda) genes could be toxic. Our research has extended these studies in a number of ways. Most significantly, we investigated the mechanism of toxicity and show it is a highly specific CRISPR/Cas-dependent process that requires Cas proteins, a PAM, particular complementarity between the protospacer and spacer in the crRNA. Furthermore, we show the toxicity can result in cellular filamentation, indicative of DNA damage, which ultimately can result in genome evolution in bacterial survivors.

For chromosomal interference to cause mutations and genome evolution, DNA must be targeted and result in toxicity. Therefore, our results are relevant for the majority of CRISPR/Cas systems, most of which are thought to target DNA. However, one subtype, the type III-B (Cmr) system, targets RNA [24]–[26] and as such is unlikely to cause toxicity/mutation, except when essential genes are targeted. The DNA damage-induced SOS response can cause higher mutation rates [63], which raises the possibility of further mutational side effects from CRISPR/Cas chromosomal targeting. The SOS response also triggers induction of many prophages, such as lambda [64], and it is interesting to speculate that chromosomal targeting might also cause phage-mediated bacterial cell suicide.

The negative impact of acquiring spacers from the chromosome suggests that mechanisms exist to avoid this. Indeed, we have shown mutations can result in tolerance to chromosomal spacers; however, this is not an efficient system and would lead to defective CRISPR/Cas systems at a high rate. In fact, this may provide some explanation as to why only half of all bacteria surveyed contain CRISPR/Cas. How do bacteria control the accidental incorporation of detrimental chromosomally-derived spacers? In experiments with the *E. coli* type I-E system, acquisition of chromosomally-derived spacers was generally PAM-dependent, but was rare relative to acquisition from a plasmid when adjusted for the number of possible PAM targets [30]. The mechanism for reducing spacer acquisition from the chromosome is unknown but is a critical question that remains to be addressed [11].

What are the mechanisms for CRISPR/Cas-mediated generation of precise (i.e. class I) and imprecise (i.e. class II) chromosomal deletions? For generation of class I mutants (i.e. **Δ**HAI2) we propose two non-mutually exclusive models; 1) as a selector of naturally occurring deletions and 2) as a sequence-specific mutator. Firstly, in the rare HAI2-excising cells (10^−6^), *attB* is formed via site-specific recombination and the excised pHAI2 will be subject to degradation due to the presence of the CRISPR/Cas target site, yielding **Δ**HAI2 strains. Secondly, CRISPR/Cas has a mutator role through chromosomal DNA damage (observed toxicity, viable counts and cell elongation), likely caused by the nuclease activity of Cas3 [65]–[67]. A double strand break (DSB) in the island, induced by CRISPR/Cas, might be repaired by precise island removal via integrase and/or excisionase-dependent site-specific recombination between *attL* and *attR*, generating *attB* (a **Δ**HAI2 strain). A linear DNA fragment would be released with *attP* flanked by any remaining DNA from either end of the island (following CRISPR degradation). This DNA would be unstable and lost upon cell division or through degradation by nucleases.

Based on our data we hypothesise a DNA degradation and alternative end joining (A-EJ) model for generation of the imprecise chromosomal deletions (i.e. class II). CRISPR/Cas targeting leads to Cas3-mediated DNA damage [65]–[67]. The Cas3 nuclease activity and/or end resection by RecBCD [68], leads to extensive degradation of host DNA. In mutants 2, 5 and 14, a region of 7 nt homology was identified at the repair site, which is too short for homologous recombination. However, A-EJ repairs DSBs by using regions of microhomology (1–9 nt) [68] and as such, could provide a model for the partial mutants.

We have developed a tool for the precise removal of genomic islands (and potentially other genomic regions) for analysis of their function in the host bacterium. During the revision of this manuscript, the type II Cas9 system was also shown to enable editing of bacterial genomes [69]. The HAI2 island that was modified in this study shares similarity in structure and sequence to the SPI-7 pathogenicity island of *Salmonella enterica* serovar Typhi [54], [70], but also contains important plant pathogenicity determinants for *P. atrosepticum* [54]. HAI2 is therefore a good example of the mosaic nature that is common in genomic islands [71]. Indeed, all class II mutants had deleted major portions of HAI2, yet retained the ability to excise and circularise (Figure 6), suggesting that CRISPR/Cas could be an important factor driving the mosaicism and evolution of genomic islands. A recent study in *Streptococcus agalactiae* demonstrated that CRISPR can inhibit acquisition of an ICE introduced by conjugation [72]. Our study shows that ICEs can be deleted or modified after their acquisition and hence, CRISPR/Cas might act at later stages to remove mobile elements from bacterial genomes. We hypothesise that upon exposure to HAI2 in the past, *P. atrosepticum* acquired a spacer targeting this ICE, but due to its integration in the chromosome it only survived due to a single nt mutation in the PAM that alleviates targeting. Other selective pressures might have existed to retain HAI2, such as the coronofacic acid pathogenicity determinants present on the island and the existence of a *pemIK* toxin-antitoxin system. The plant host can also influence the loss or transfer and selection for genomic islands, as exemplified by the *in planta* dynamics of the PPHGI-1 island in *Pseudomonas syringae* pv. *phaseolicola* [73], [74]. HAIs are important in the evolution of bacteria, due to their transfer of virulence genes or other ecologically important traits [75] and are considered part of the accessory genome, which can constitute 10% of the entire chromosome [76]. A simple method for positive selection of their deletion promises advances into the study of their functional roles.

In conclusion, we demonstrated that chromosomal targeting is highly detrimental to bacterial growth and that mutations that inactivate the CRISPR/Cas systems allow survival. The negative impact of this targeting resulted in the selection of strains containing dramatic mutations in the target site, such as those deleted for part of (e.g. ∼40 kb), or an entire (∼100 kb) pathogenicity island. Therefore, CRISPR/Cas systems can play a significant role in the evolution of bacterial genomes that may influence pathogenicity. We propose that chromosomal targeting has resulted in widespread changes to bacterial genomes, a prediction prompting further bioinformatic studies.

## Materials and Methods

### Bacterial strains and growth conditions

All strains and plasmids used in this study are given in Table S1 and Table S2, respectively. *P. atrosepticum* SCRI1043 [54] was grown at 25°C and *E. coli* at 37°C in Luria Broth (LB) at 180 rpm or on LB-agar plates containing 1.5% (w/v) agar. When required, medium was supplemented with the following: ampicillin (Ap; 100 µg/ml), chloramphenicol (Cm; 25 µg/ml), kanamycin (Km; 50 µg/ml), tetracycline (Tc; 10 µg/ml), D-glucose (0.2% w/v) and L-arabinose (0.1% w/v). Bacterial growth was measured in a Jenway 6300 spectrophotometer at 600 nm (OD_600_). All experiments were repeated in at least three biological replicates.

### Molecular biology and DNA sequencing

All oligonucleotides are from Invitrogen or IDT and are listed in Table S4. All strains and plasmids were confirmed by PCR and DNA sequencing was performed at the Allan Wilson Centre, NZ. Plasmid DNA was prepared using Zyppy Plasmid Miniprep Kits (Zymo Research) according to manufacturer's guidelines. DNA from PCR and agarose gels was purified using the GE Healthcare Illustra GFX PCR DNA and Gel Band Purification Kit. Restriction enzymes and T4 ligase were from Roche or NEB.

### Construction of engineered CRISPR arrays

Plasmids were designed that would enable the cloning of any spacer and repeat sequence into an artificial CRISPR1 array that contains one repeat, yielding a CRISPR with two repeats and one spacer. The enzyme BbsI was chosen because it cuts at a distance from its recognition site and hence, it was used to cut in the repeat and could be used multiple times to introduce subsequent spacer-repeat sequences (Figure 1). Firstly, either 780, 180, 52 or 16 bp of the CRISPR1 leader and first repeat region was amplified with forward primers AM05, TGO9, RVO1 or RVO2 and the reverse primer TGO10, the products were digested with XmaI and SalI and ligated to pBAD30 previously digested with the same enzymes. The resulting CRISPR spacer entry plasmids were named pC1-780, pC1-180, pC1-52 and pC1-16 (Table S3). These plasmids were then digested with BbsI and products derived from the primer pairs that contained engineered spacer-repeat units (Table S1 and Table S4) were cloned into this site to incorporate one exact new spacer and repeat. This procedure was repeated up to three times, but could in theory be performed indefinitely to construct artificial arrays.

### *P. atrosepticum* transformation assays

Electrocompetent *P. atrosepticum* cells were prepared as follows. Ten ml *P. atrosepticum* cultures were grown overnight in LB and used to inoculate flasks of LB, which were grown until the OD_600_ reached 0.4–0.6. The cells were pelleted by centrifugation at 4°C at 2219×*g*, resuspended in ice-cold H_2_O and centrifuged as above. The H_2_O wash was repeated and then cells were washed using ice-cold 10% glycerol. Cells were resuspended in 10% glycerol, divided into 50 µl aliquots and stored at −80°C. For transformations, DNA was adjusted to working concentrations of 50 ng/µl ±5 ng/µl and 50 ng was added to 50 µl of competent *P. atrosepticum* cells. The cells and DNA were incubated on ice for 10 min then electroporated (1 mm electro-cuvettes, 1800 V, capacitance 25 µF and resistance 200 ohms). Bacteria were recovered in 1 ml LB for 2 h at 25°C and then plated on LB containing the appropriate supplements and grown at 25°C. Transformation efficiency was calculated as transformants/ng of DNA and normalised to control plasmids.

### Controlled induction of spacer arrays

Strains containing inducible pBAD30-based plasmids expressing single spacers from CRISPR1 arrays that targeted *lacZ*, *expI* or *eca0560* were used to inoculate 10 ml LB containing 0.2% glucose and Ap and incubated overnight. These cultures were used to inoculate individual 25 ml cultures of LB with either 0.2% glucose or 0.2% arabinose (in 250 ml flasks) at a starting OD_600_ of 0.05. Cultures were grown at 25°C with shaking at 180 rpm and growth (OD_600_) was measured. Where indicated, the viable count was determined by measuring the cfu/ml. Briefly, 1 ml samples of culture were taken, serially diluted and 10 µl of each dilution was plated onto LB agar containing Ap and 0.2% glucose. The plates were incubated at 25°C and colonies were counted.

### Construction of an *expI* deletion strain

A plasmid for the construction of an *expI* deletion mutant was generated as follows. Firstly, approximately 500 bp of sequence 5′ and 3′ of *expI* was amplified by PCR using primer pairs PF314 and PF315 and PF316 and PF317. Secondly, these products were used as template in an overlap extension PCR with primers PF314 and PF317. This product was digested with KpnI and XbaI and cloned into pBluescriptII SK+ cut with the same enzymes, yielding plasmid pTA163. Next, the *cat* gene and promoter were amplified by PCR using primers TGO74 and TGO75 with pACYC184 as template. This product was digested with HindIII and SalI and ligated with pTA163 cut with the same enzymes, giving plasmid pTA164. Finally, the 5′ 500 bp-*cat*-500 bp 3′ fragment was subcloned from pTA164 into pKNG101 on a BamHI/XbaI fragment, yielding plasmid pTA165. *E. coli* CC118λpir carrying pTA165 was used in an allelic exchange protocol as previously described [77], [78] via triparental mating with the helper *E. coli* strain HH26, pNJ5000 and *P. atrosepticum* as the recipient, resulting in strain PCF81 (Δ*expI*::*cat*).

### Construction of strains with reintroduced variant protospacers

Engineered variant protospacers were introduced into *P. atrosepticum* as described below. Plasmid pTA164 was used as the template in a PCR with RV19 and PF317 to amplify a 1.5 kb fragment containing the *cat* gene and the 500 bp 3′ *expI* flanking sequence. RV19 contained the protospacer sequence for the *expI-*1 spacer with a 5′-protospacer-GG-3′ PAM. The resulting PCR fragment was cloned using XhoI and XbaI restriction sites into pTA164 to make pRX38. To generate other protospacer variants, forward primers that introduced the *expI*-1 protospacer sequence with a 5′-protospacer-TG-3′ PAM (RV20), a C6T mutation (RV21) or a C3T mutation (RV29) were used in PCRs with PF317 and pRX38 as template. The resulting PCR fragments were cloned using XhoI and XbaI restriction sites into pTA164 to make plasmids pRX39 (5′-protospacer-TG-3′ PAM), pRX40 (C6T) and pRX34 (C3T). Finally, the 2 kb BamHI/XbaI 5′ 500 bp-*cat*-500 bp 3′ fragments containing the variant protospacers from pRX38, pRX39, pRX40 and pRX34 were cloned into pKNG101 to make pRX41, pRX42, pRX43 and pRX35, respectively. Allelic exchange was performed with these suicide plasmids, as described above for PCF81, and resulted in strains RBV01 (GG PAM), RBV02 (5′-protospacer-TG-3′ PAM), RBV03 (C6T) and RBV04 (C3T).

### LIVE/DEAD staining, fluorescence microscopy, and TEM

The LIVE/DEAD BacLight Bacterial Viability Kit (Invitrogen) was used to stain cells, which were then observed with an Olympus BX51 microscope. Samples were pelleted at 13000 rpm for 5 min then resuspended in distilled water and pelleted again to wash away the growth media at least once and finally resuspended in 500 µl of distilled water. One hundred µl of washed culture (approximately 1×10^7^ cells) was taken and stained with 1.5 µl of the flurophore SYTO9 (green fluorescent, 480–500 nm), and 1.5 µl of propidium iodide (PI) (red fluorescent, 490–635 nm). Cells were stained for 20 min in the dark, 8 µl samples were mounted on slides and imaged under the oil immersion 100× lens. For TEM, carbon-coated copper grids were prepared using standard methods as previously described [79]. Grids were analysed using the Phillips CM100 BioTWIN transmission electron microscope. Cell measurements were performed on TEM images of at least 60 randomly selected cells from each treatment.

### Site-directed mutagenesis of CRISPR1 repeats flanking the *expI-*1 spacer

The CRISPR1 repeat mutations, G20A, C18A and C18A/G8U were introduced into the sequence of the pE1-16 array using primer pairs RV22 and RV25, RV23 and RV26 and RV24 and RV27, respectively. Products were amplified by PCR with 8% DMSO due to the CRISPR repeat secondary structures in primers RV22, RV23 and RV24. These products were cloned into pBAD30 with XmaI and BbsI making the plasmids, pE1-16 G20A, pE1-16 C18A, and pE1-16 C18A/G8U, respectively.

### Loss and deletion of HAI2 by CRISPR/Cas–mediated targeting

A Km^R^ transposon mutation in *eca0573* on HAI2 (JTC101) was isolated in an independent random mutagenesis (Chang and Fineran; unpublished data) and provided a marker to screen for island loss. Strains of JTC101 with either a vector control (pBAD30) or plasmids targeting *expI* (pE1-16) or *eca0560* (pTraG1-16) were grown in 10 ml LB with Ap and 0.2% arabinose for 36 h with Ap added to the media at 12 h intervals to ensure plasmid maintenance. By plating on LBA with Ap and 0.2% arabinose we isolated approximately 600 colonies from at least 12 independent cultures per strain that were resistant to CRISPR/Cas-associated HAI2 targeting. These survivors were patched onto LBA plates containing Ap and 0.2% arabinose with or without Km. Mutants that failed to grow on the plates containing Km were isolated and subjected to PCR to check for loss of the transposon and to ascertain if parts of HAI2 were lost due to CRISPR/Cas targeting. The same approach was used for targeting of *expI* using plasmid pE1-16 in strain RBV01, which contains a single expI protospacer immediately adjacet to a Cm^R^ cassette. In addition, *lacZ* was targeted by plasmid pL1-16 in the WT background. The primers used are listed on Table S4 and details are given in the results.

## Supporting Information

Figure S1 Transformation of *P. atrosepticum* with plasmids expressing spacers with chromosomal targets does not affect the transformation efficiency. A) WT and B) Δ*cas* strains were transformed with plasmids with 780 bp of leader sequence and containing no spacers (pC1-780), 3 scrambled spacers (pNS3-780) or 3 spacers targeting *expI* (pE3-780). The average transformation efficiency in cfu/ng plasmid DNA is shown ± SD of three biological replicates.
(TIF)

Figure S2 Generation of controllable CRISPR plasmids and identification of a putative CRISPR1 promoter. Leader truncations of CRISPR1 containing three anti-*expI* spacers from A) 780 (pE3-780), B) 180 (pE3-180), C) 52 (pE3-52) to D) 16 bp (pE3-16) were generated and transformed into WT and Δ*cas* (PCF80) strains and plated on LBA with Ap and 0.2% glucose to repress *P_araBAD_* expression. Representative plates are shown from experiments performed in at least biological triplicates. The same effect was observed with leader truncations of CRISPR1 containing three anti-*lacZ* spacers from 780 (pL3-780), 180 (pL3-180), 52 (pL3-52) to 16 bp (pL3-16) when transformed into WT and Δ*cas* strains (data not shown). E) Leader sequence present in the 52 and 16 bp leader constructs and the predicted −35 and −10 promoter elements (blue) relative to the first CRISPR repeat (red).
(TIF)

Figure S3 *In vitro* processing of pre-crRNA from CRISPR2. (A) *In vitro* processing assay of ^32^P-uniformly labelled CRISPR2 substrates that contain two repeats and span either spacer 2 or spacer 6. Soluble protein fractions were generated from a Δ*cas* mutant (PCF80), a Δ*cas* strain expressing Cas6f (pJSC6) and WT *P. atrosepticum*. –protein is the negative control in the absence of any soluble cell extract. An *in vitro* transcript of the Hammerhead ribozyme sequence of *Arabidopsis thaliana*, which specifically cleaves itself during the transcription reaction (HHRz), was used as ladder. The spacer 2 and spacer 6 regions were amplified using primer pairs RP46 and RP47 and RP44 and RP45 and cloned into pGEM-Teasy (Promega) giving pRP19 and pRP20. Cas6f CRISPR RNA processing assays were performed as described previously [23]. (B) Schematic representation of the probe design (top), where green indicates the spacer (either 2 or 6), black indicates repeats and yellow and grey indicate partial adjacent spacers. The wavy line indicates some vector sequence present after *in vitro* transcription. Cleavage products are indicated and aligned with the bands detected in the gel shown in (A). Products resulting from single and double endonuclease cleavage events were detected for both spacer 2 and spacer 6 CRISPR2 substrates.
(TIF)

Figure S4 Screening of HAI2 mutants for partial or complete loss of the island. (A) Mutants that conferred kanamycin sensitivity (Km^S^) after CRISPR-directed targeting of the *eca0560* gene were isolated. The *attB* junction in each mutant was amplified by colony PCR to determine the precise excision or partial loss of the HAI2. Positive bands indicate the precise excision of the island (class I mutants), whereas negative bands indicate HAI2 is retained but has undergone partial deletions (class II mutants). (B) Presence and excision of the HAI2 derivatives in the 7 class II mutants was further confirmed by the amplification of the *attL*, *attR* and *attP* junctions by colony PCR. (C) Sequence of the reconstituted 49 bp *attB* junction after HAI2 excision (co-ordinates refer to the published *P. atrosepticum* SCRI1043 genome). Primers used are listed in Table S4.
(TIF)

Figure S5 Mapping the partial deletion of HAI2 class II mutants. (A) Positive PCR controls were performed to indicate the presence of selected genes spanning throughout HAI2. (B–F) To determine the extent of the deletions within HAI2, colony PCR for specified genes was performed for each mutant. PCR profiles of the seven classified HAI2 class II mutants are presented. For (C), only the profile for mutant 2 is shown as mutants 5 and 14 share the same profile. Amplification of the *cas1* gene outside of HAI2 acted as a PCR positive control for all mutants. PCR products amplifying specified gap junctions were sequenced to accurately determine the site of deletion. A graphical overview of the results is shown in Figure 6E and primers used are listed in Table S4.
(TIF)

Figure S6 Mapping chromosomal deletions following targeting of *lacZ*. (A) Genome organization and mapping of deleted areas after CRISPR-directed targeting of the *lacZ* gene (blue). Black boxes are areas amplified by colony PCR to confirm the presence or absence of specified genes. Roughly, a chromosomal deletion of greater than 50 kb including the *lacZ* gene and two NRPSs was detected. (B) Ten *lacZ* mutants isolated were subjected to colony PCR to determine sites of deletions as indicated in the above diagram. Primers used are listed in Table S4.
(TIF)

Table S1 CRISPR spacers used in this study.
(PDF)

Table S2 Bacterial strains and bacteriophage used in this study.
(PDF)

Table S3 Plasmids used in this study.
(PDF)

Table S4 Oligonucleotide primers used in this study.
(PDF)
