# Supplementary figures and images for: Cytotoxic Chromosomal Targeting by CRISPR/Cas Systems Can Reshape Bacterial Genomes and Expel or Remodel Pathogenicity Islands
Source: PLoS Genet. 2013 Apr 18;9(4):e1003454. doi: 10.1371/journal.pgen.1003454 (PMC3630108; doi:10.1371/journal.pgen.1003454)

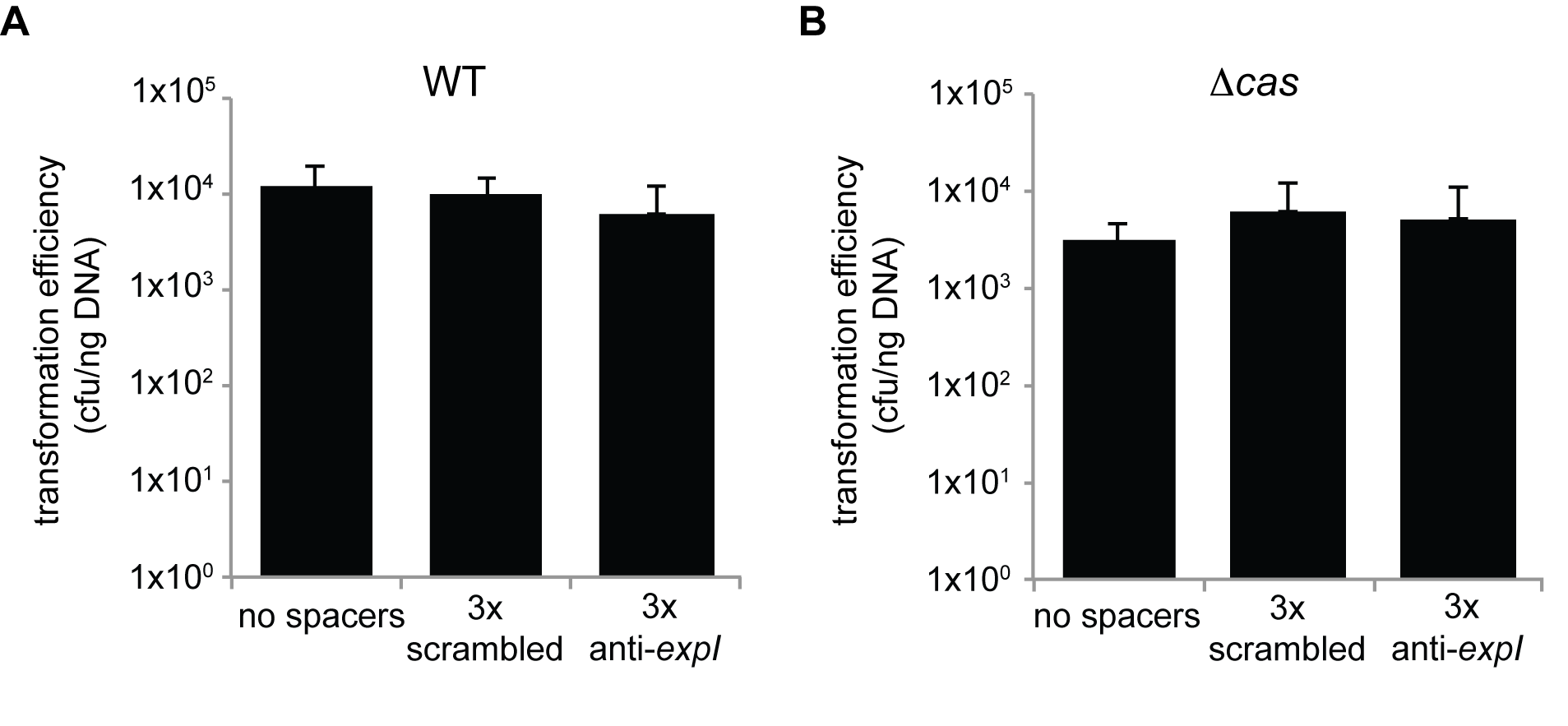

Supplement: Figure S1 — Transformation of P. atrosepticum with plasmids expressing spacers with chromosomal targets does not affect the transformation efficiency. A) WT and B) Δcas strains were transformed with plasmids with 780 bp of leader sequence and containing no spacers (pC1-780), 3 scrambled spacers (pNS3-780) or 3 spacers targeting expI (pE3-780). The average transformation efficiency in cfu/ng plasmid DNA is shown ± SD of three biological replicates. (TIF) [file pgen.1003454.s001.tif]

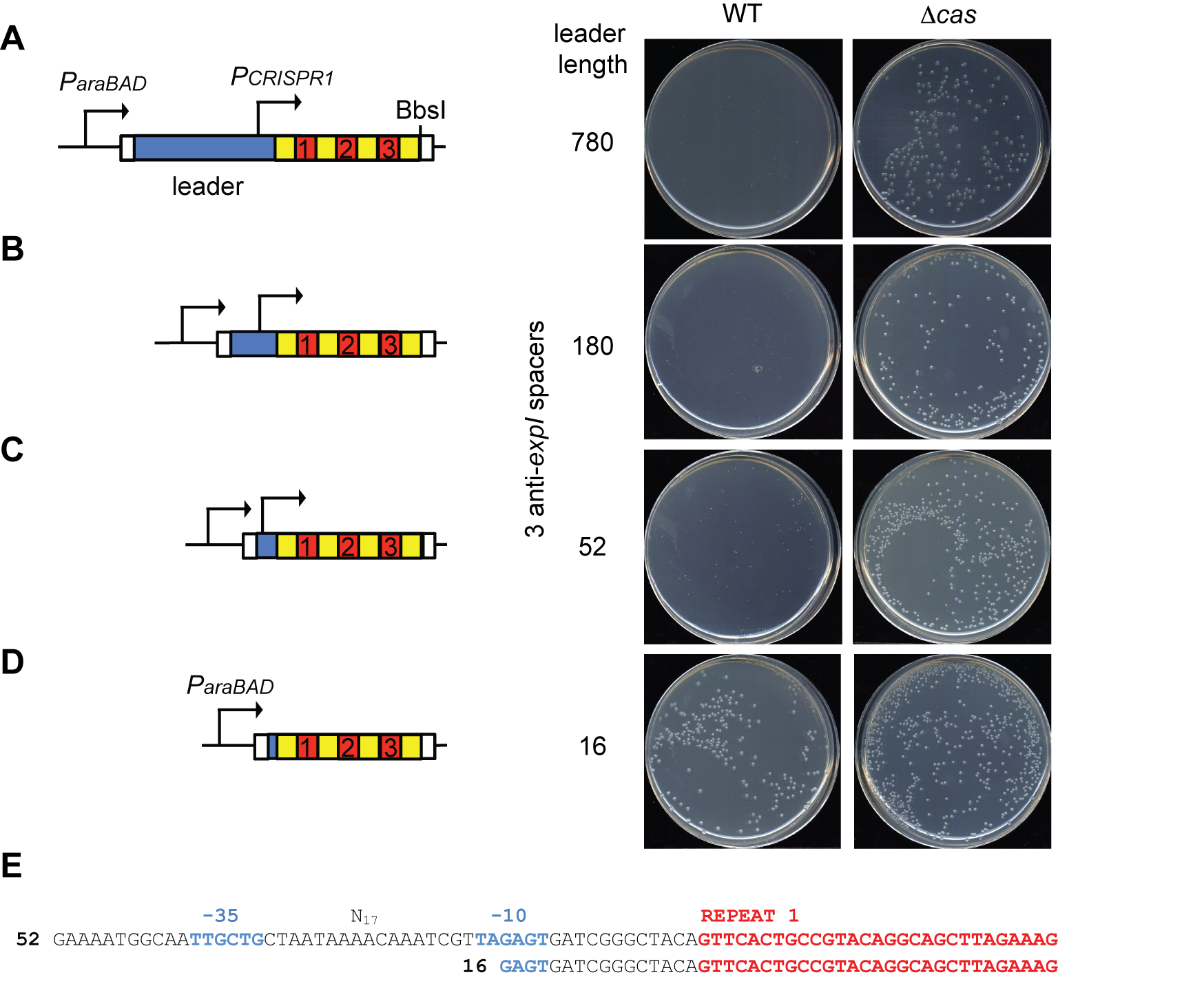

Supplement: Figure S2 — Generation of controllable CRISPR plasmids and identification of a putative CRISPR1 promoter. Leader truncations of CRISPR1 containing three anti-expI spacers from A) 780 (pE3-780), B) 180 (pE3-180), C) 52 (pE3-52) to D) 16 bp (pE3-16) were generated and transformed into WT and Δcas (PCF80) strains and plated on LBA with Ap and 0.2% glucose to repress ParaBAD expression. Representative plates are shown from experiments performed in at least biological triplicates. The same effect was observed with leader truncations of CRISPR1 containing three anti-lacZ spacers from 780 (pL3-780), 180 (pL3-180), 52 (pL3-52) to 16 bp (pL3-16) when transformed into WT and Δcas strains (data not shown). E) Leader sequence present in the 52 and 16 bp leader constructs and the predicted −35 and −10 promoter elements (blue) relative to the first CRISPR repeat (red). (TIF) [file pgen.1003454.s002.tif]

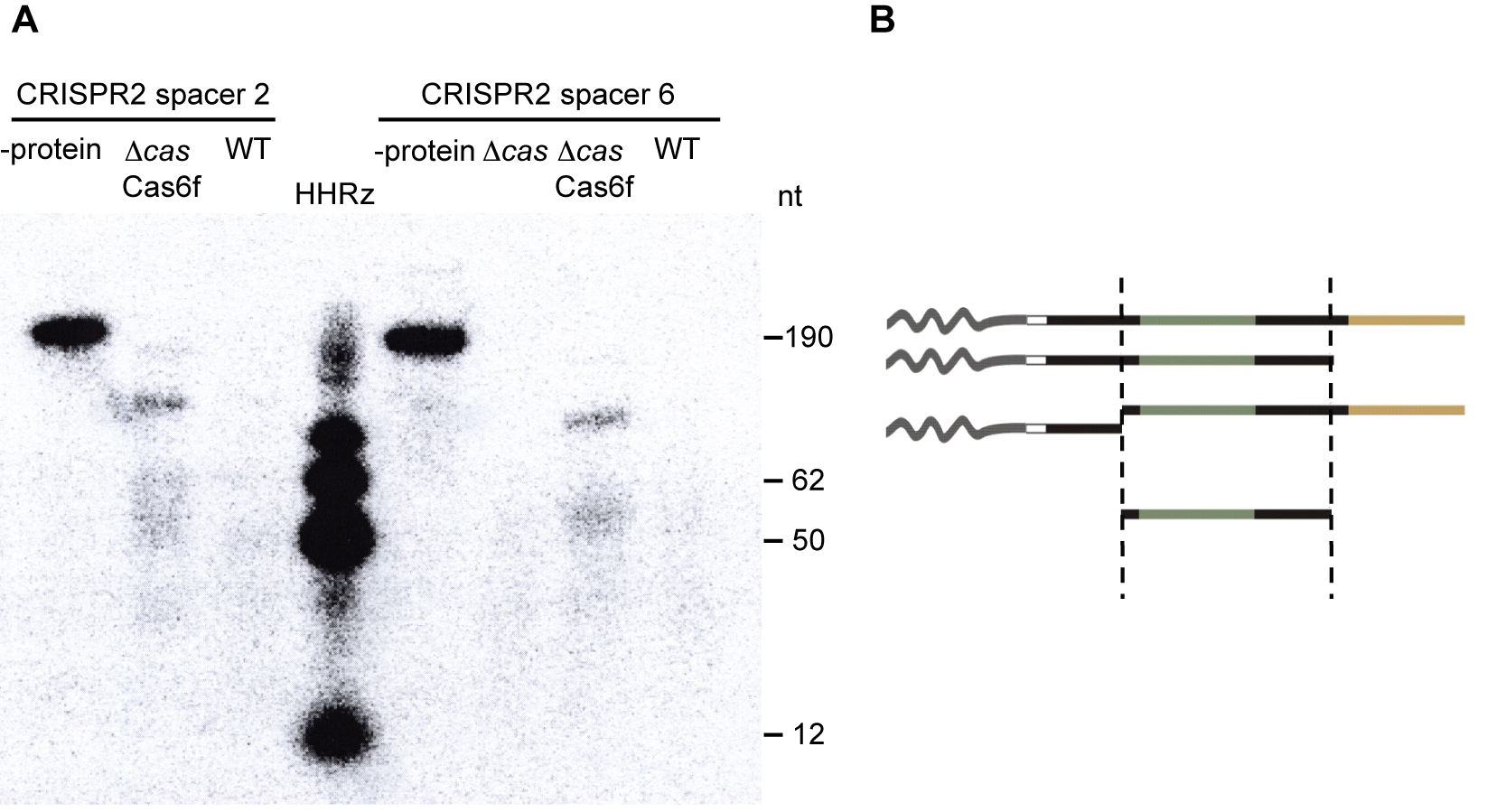

Supplement: Figure S3 — In vitro processing of pre-crRNA from CRISPR2. (A) In vitro processing assay of 32P-uniformly labelled CRISPR2 substrates that contain two repeats and span either spacer 2 or spacer 6. Soluble protein fractions were generated from a Δcas mutant (PCF80), a Δcas strain expressing Cas6f (pJSC6) and WT P. atrosepticum. –protein is the negative control in the absence of any soluble cell extract. An in vitro transcript of the Hammerhead ribozyme sequence of Arabidopsis thaliana, which specifically cleaves itself during the transcription reaction (HHRz), was used as ladder. The spacer 2 and spacer 6 regions were amplified using primer pairs RP46 and RP47 and RP44 and RP45 and cloned into pGEM-Teasy (Promega) giving pRP19 and pRP20. Cas6f CRISPR RNA processing assays were performed as described previously [23]. (B) Schematic representation of the probe design (top), where green indicates the spacer (either 2 or 6), black indicates repeats and yellow and grey indicate partial adjacent spacers. The wavy line indicates some vector sequence present after in vitro transcription. Cleavage products are indicated and aligned with the bands detected in the gel shown in (A). Products resulting from single and double endonuclease cleavage events were detected for both spacer 2 and spacer 6 CRISPR2 substrates. (TIF) [file pgen.1003454.s003.tif]

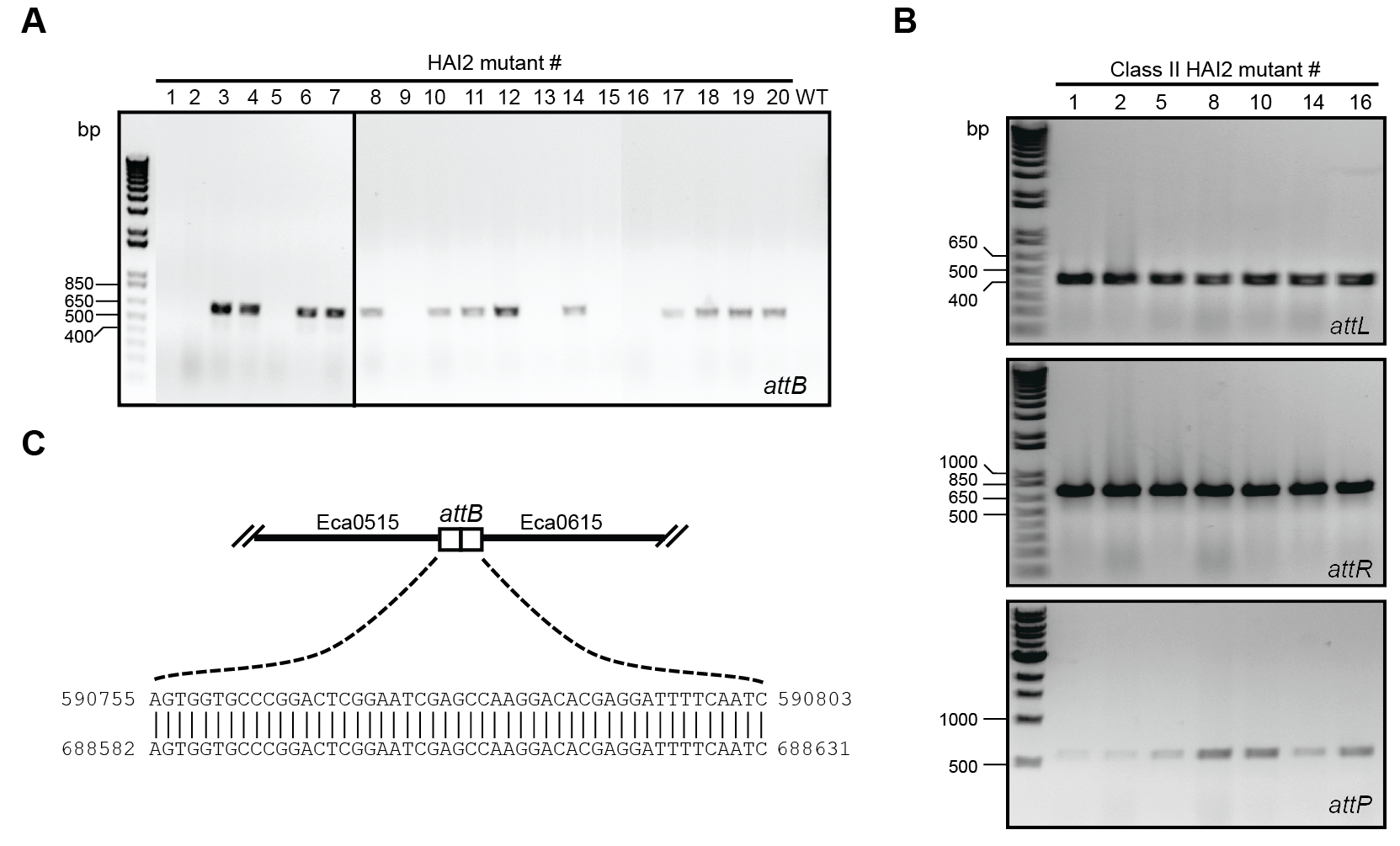

Supplement: Figure S4 — Screening of HAI2 mutants for partial or complete loss of the island. (A) Mutants that conferred kanamycin sensitivity (KmS) after CRISPR-directed targeting of the eca0560 gene were isolated. The attB junction in each mutant was amplified by colony PCR to determine the precise excision or partial loss of the HAI2. Positive bands indicate the precise excision of the island (class I mutants), whereas negative bands indicate HAI2 is retained but has undergone partial deletions (class II mutants). (B) Presence and excision of the HAI2 derivatives in the 7 class II mutants was further confirmed by the amplification of the attL, attR and attP junctions by colony PCR. (C) Sequence of the reconstituted 49 bp attB junction after HAI2 excision (co-ordinates refer to the published P. atrosepticum SCRI1043 genome). Primers used are listed in Table S4. (TIF) [file pgen.1003454.s004.tif]

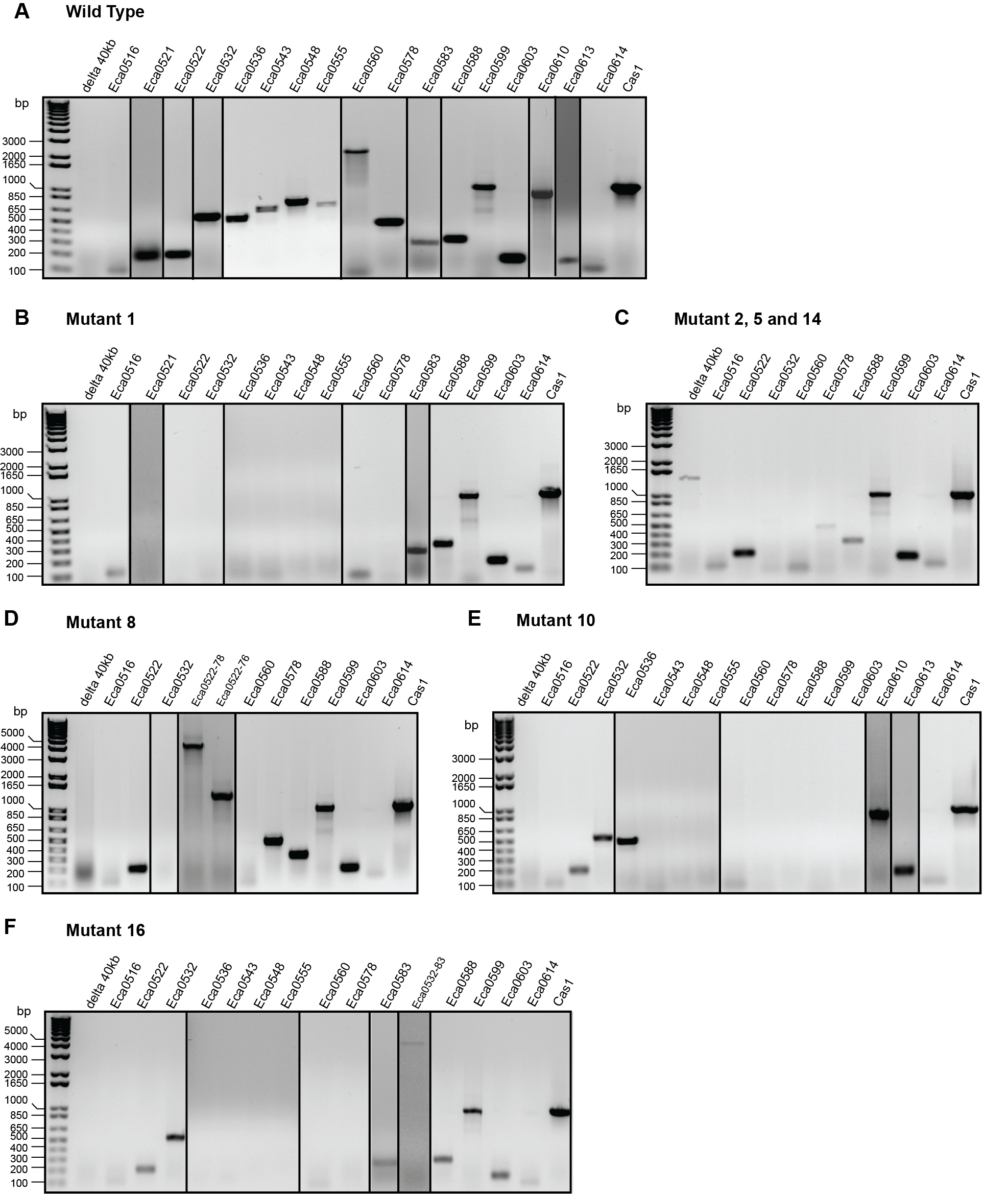

Supplement: Figure S5 — Mapping the partial deletion of HAI2 class II mutants. (A) Positive PCR controls were performed to indicate the presence of selected genes spanning throughout HAI2. (B–F) To determine the extent of the deletions within HAI2, colony PCR for specified genes was performed for each mutant. PCR profiles of the seven classified HAI2 class II mutants are presented. For (C), only the profile for mutant 2 is shown as mutants 5 and 14 share the same profile. Amplification of the cas1 gene outside of HAI2 acted as a PCR positive control for all mutants. PCR products amplifying specified gap junctions were sequenced to accurately determine the site of deletion. A graphical overview of the results is shown in Figure 6E and primers used are listed in Table S4. (TIF) [file pgen.1003454.s005.tif]

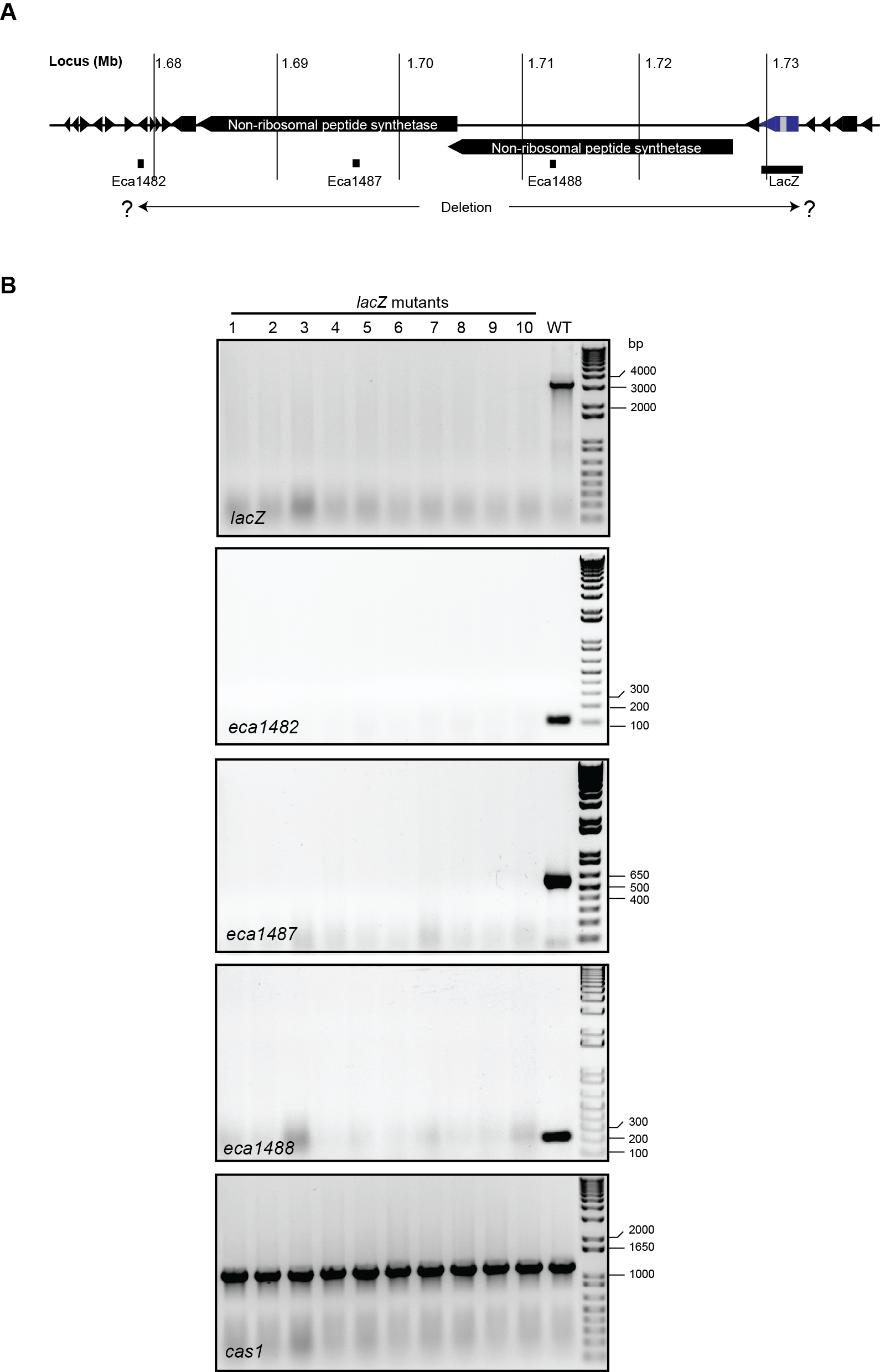

Supplement: Figure S6 — Mapping chromosomal deletions following targeting of lacZ. (A) Genome organization and mapping of deleted areas after CRISPR-directed targeting of the lacZ gene (blue). Black boxes are areas amplified by colony PCR to confirm the presence or absence of specified genes. Roughly, a chromosomal deletion of greater than 50 kb including the lacZ gene and two NRPSs was detected. (B) Ten lacZ mutants isolated were subjected to colony PCR to determine sites of deletions as indicated in the above diagram. Primers used are listed in Table S4. (TIF) [file pgen.1003454.s006.tif]
