# Supplementary material for: Cytotoxic Chromosomal Targeting by CRISPR/Cas Systems Can Reshape Bacterial Genomes and Expel or Remodel Pathogenicity Islands
Source: PLoS Genet. 2013 Apr 18;9(4):e1003454. doi: 10.1371/journal.pgen.1003454 (PMC3630108; doi:10.1371/journal.pgen.1003454)
Supplement: Table S1 — CRISPR spacers used in this study. (PDF) [file pgen.1003454.s007.pdf]

Table S1. CRISPR spacers used in this study

**Spacer**	**Sequence 5′-3′**	**Primers**	**PAM** 5′-protospacer-PAM-3′*
scrambled 1	ACATCACACGAATCTTATGCTTGTGATGGTCG	TG012 & TG013	Not applicable
scrambled 2	CAGTACACGGTATGGATGCTGATGCTGAACTG	TG014 & TG015	Not applicable
scrambled 3	ACTAAGTCCACTGAAACGTCTGAAGGCGTAAT	TG016 & TG017	Not applicable
anti-*expI* 1	TTGTTGTCAGAAACGAAATCAGAAGAGCTATT	PF273 & PF274	GG
anti-*expI* 2	TCAGAAAAGAGACGTTTAAAGATCGACTGAAT	PF275 & PF276	GG
anti-*expI* 3	TATGCTAACGATATTAAAACGTTCTGGCTGGG	PF277 & PF278	GG
anti-*lacZ* 1	GTTACGTTGGTCTGACGGAAGTTATCTGGAAG	TG054 & TG055	AC
anti-*lacZ* 2	CTGGATAACGACATCGGTATCAGCGAAGTCGA	TG056 & TG057	TG
anti-*lacZ* 3	TACAGCGAACGCGTATCAAGGATGGTGCAGCG	PF279 & PF280	GG
anti-*eca0560* 1	TCCAGTACTCAGGATCGTGTTGGTACGATAAA	PF326 & PF327	TG
anti-*eca0560* 2	TTCAGTCAGTTATGTGACGGCCGGTGGGTTCG	RV14 & RV15	GG

*PAM is designated on the targeted strand.
