# Supplementary material for: Cytotoxic Chromosomal Targeting by CRISPR/Cas Systems Can Reshape Bacterial Genomes and Expel or Remodel Pathogenicity Islands
Source: PLoS Genet. 2013 Apr 18;9(4):e1003454. doi: 10.1371/journal.pgen.1003454 (PMC3630108; doi:10.1371/journal.pgen.1003454)
Supplement: Table S2 — Bacterial strains and bacteriophage used in this study. (PDF) [file pgen.1003454.s008.pdf]

Table S2. Bacterial strains and bacteriophage used in this study.

Strain/Plasmid	Genotype/Phenotype	Reference
Escherichia coli		
CC118 λ*pir*	*araD*, Δ(*ara*, *leu*), Δ*lacZ*74, *phoA*20, *galK*, *thi-1*, *rspE*, *rpoB*, *argE*, *recA*1, λpir	[1]
DH5α	F^-, ϕ80Δd*lacZ*M15, Δ(*lacZYA–argF*)U169, *endA1*, *recA1*, *hsd*R17 ($r_K^- m_K^+$), *deoR*, *thi-1*, *sup*E44, λ^-, *gyr*A96, *rel*A1	Gibco/BRL
HH26	Marker exchange mobilisation strain for conjugal transfer	[2]
SM10 λ*pir*	*thi-1*, *thr*, *leu*, *tonA*, *lacY*, *supE*, *recA*::RP4-2-Tc::Mu, λ*pir*, Km^R	[3]
Pectobacterium atrosepticum		
JTC101	*eca0573*::Tn-DS1028-*uidA*Km, Km^R	This study
PCF79	Δ*cas13csy1234*::*lacZcat* (operon deletion, *lacZ* translational fusion), Cm^R	[4]
PCF80	Δ*cas13csy1234*::*cat* (operon deletion), Cm^R	[4]
PCF81	Δ*expI*::*cat* (deletion), Cm^R	This study
PCF82	Δ*csy4*::*cat* (deletion), Cm^R	[4]
RBV01	Δ*expI*::*cat* containing anti-*expI*1 protospacer and 5′-protospacer-GG-3′ PAM, Cm^R	This study
RBV02	Δ*expI*::*cat* containing anti-*expI*1 protospacer and 5′-protospacer-TG-3′ PAM, Cm^R	This study
RBV03	Δ*expI*::*cat* containing anti-*expI*1 C6T protospacer and 5′-protospacer-GG-3′ PAM, Cm^R	This study
RBV04	Δ*expI*::*cat* containing anti-*expI*1 C3T protospacer and 5′-protospacer-GG-3′ PAM, Cm^R	This study
SCRI1043	Wild type (WT)	[5]
Phage		
ϕOT8S	*Serratia* sp. strain ATCC39006 phage; scrambled sequence template	unpublished

References

1. Herrero M, de Lorenzo V, Timmis KN (1990) Transposon vectors containing non-antibiotic resistance selection markers for cloning and stable chromosomal insertion of foreign genes in gram-negative bacteria. J Bacteriol 172: 6557-6567.
2. Grinter NJ (1983) A broad-host-range cloning vector transposable to various replicons. Gene 21: 133-143.
3. de Lorenzo V, Herrero M, Jakubzik U, Timmis KN (1990) Mini-Tn5 transposon derivatives for insertion mutagenesis, promoter probing, and chromosomal insertion of cloned DNA in gram-negative eubacteria. J Bacteriol 172: 6568-6572.
4. Przybilski R, Richter C, Gristwood T, Clulow JS, Vercoe RB, et al. (2011) Csy4 is responsible for CRISPR RNA processing in *Pectobacterium atrosepticum*. RNA Biol 8: 517-528.
5. Bell KS, Sebaihia M, Pritchard L, Holden MT, Hyman LJ, et al. (2004) Genome sequence of the enterobacterial phytopathogen *Erwinia carotovora* subsp. *atroseptica* and characterization of virulence factors. Proc Natl Acad Sci U S A 101: 11105-11110.
