# Supplementary material for: Cytotoxic Chromosomal Targeting by CRISPR/Cas Systems Can Reshape Bacterial Genomes and Expel or Remodel Pathogenicity Islands
Source: PLoS Genet. 2013 Apr 18;9(4):e1003454. doi: 10.1371/journal.pgen.1003454 (PMC3630108; doi:10.1371/journal.pgen.1003454)
Supplement: Table S3 — Plasmids used in this study. (PDF) [file pgen.1003454.s009.pdf]

Table S3. Plasmids used in this study.

Plasmid	Genotype/Phenotype	Reference
Plasmids for engineered CRISPR arrays		
pBAD30	Arabinose inducible expression vector, Ap^R	[1]
pC1-780	CRISPR1 with 780 bp leader and no spacers, Ap^R	This study
pS3-780	CRISPR1 with 780 bp leader and scrambled spacers 1, 2 and 3, Ap^R	This study
pL3-780	CRISPR1 with 780 bp leader and *lacZ* spacers 1, 2 and 3, Ap^R	This study
pE3-780	CRISPR1 with 780 bp leader and *expI* spacers 1, 2 and 3, Ap^R	This study
pC1-180	CRISPR1 with 180 bp leader and no spacers, Ap^R	This study
pS3-180	CRISPR1 with 180 bp leader and scrambled spacers 1, 2 and 3, Ap^R	This study
pL3-180	CRISPR1 with 180 bp leader and *lacZ* spacers 1, 2 and 3, Ap^R	This study
pE3-180	CRISPR1 with 180 bp leader and *expI* spacers 1, 2 and 3, Ap^R	This study
pC1-52	CRISPR1 with 52 bp leader and no spacers, Ap^R	This study
pS3-52	CRISPR1 with 52 bp leader and scrambled spacers 1, 2 and 3, Ap^R	This study
pL3-52	CRISPR1 with 52 bp leader and *lacZ* spacers 1, 2 and 3, Ap^R	This study
pE3-52	CRISPR1 with 52 bp leader and *expI* spacers 1, 2 and 3, Ap^R	This study
pC1-16	CRISPR1 with 16 bp leader and no spacers, Ap^R	This study
pS3-16	CRISPR1 with 16 bp leader and scrambled spacers 1, 2 and 3, Ap^R	This study
pL3-16	CRISPR1 with 16 bp leader and *lacZ* spacers 1, 2 and 3, Ap^R	This study
pL1-16	CRISPR1 with 16 bp leader and *lacZ* spacer 3	This study
pE3-16	CRISPR1 with 16 bp leader and *expI* spacers 1, 2 and 3, Ap^R	This study
pE1-16	CRISPR1 with 16 bp leader and *expI* spacer 1, Ap^R	This study
pTraGS6-16	CRISPR1 with 16 bp leader and CRISPR2 spacer 6, Ap^R	This study
pTraG1-16	CRISPR1 with 16 bp leader and *eca0560* spacer 1, Ap^R	This study
pTraG2-16	CRISPR1 with 16 bp leader and 2× *eca0560* spacer 1, Ap^R	This study
pTraG3-16	CRISPR1 with 16 bp leader and 3× *eca0560* spacer 1, Ap^R	This study
pTraG8-16	CRISPR1 with 16 bp leader and 8× *eca0560* spacer 1, Ap^R	This study
pE1-16 G20A	CRISPR1 with 16 bp leader, *expI* spacer 1 and G20A repeat mutation, Ap^R	This study
pE1-16 C18A	CRISPR1 with 16 bp leader, *expI* spacer 1 and C18A repeat mutation, Ap^R	This study
pE1-16 C18A/G8U	CRISPR1 with 16 bp leader, *expI* spacer 1 and C18A/G8U repeat mutations, Ap^R	This study
Plasmids for Δ*expI,* protospacer and PAM mutant constructions		
pACYC184	Cloning vector, p15A replicon, Cm^R, Tet^R	[2]
pBluescript II SK+	Cloning vector (pBS II SK+), ColE1 replicon, Ap^R	Stratagene
pTA163	Δ*expI* intermediate in pBS II SK+, Ap^R	This study
pTA164	Δ*expI*::*cat* intermediate in pBS II SK+, Ap^R, Cm^R	This study
pKNG101	Marker exchange suicide vector, *sacBR*, *mobRK2*, *oriR6K*, Sm^R	[3]
pNJ5000	Mobilising plasmid used in marker exchange, Tc^R	[4]
pTA165	Δ*expI*::*cat* for allelic exchange in pKNG101, Cm^R, Sm^R	This study
pRX34	C3T *expI*-1 Δ*expI*::*cat* intermediate in pBS II SK+, Ap^R, Cm^R	This study
pRX35	C3T *expI*-1 Δ*expI*::*cat* for allelic exchange in pKNG101, Cm^R, Sm^R	This study
pRX38	5′-protospacer-GG-3′ PAM *expI*-1 Δ*expI*::*cat* intermediate in pBS II SK+, Ap^R, Cm^R	This study
pRX39	5′-protospacer-TG-3′ PAM *expI*-1 Δ*expI*::*cat* intermediate in pBS II SK+, Ap^R, Cm^R	This study
pRX40	C6T *expI*-1 Δ*expI*::*cat* intermediate in pBS II SK+, Ap^R, Cm^R	This study
pRX41	5′-protospacer-GG-3′ PAM *expI*-1 Δ*expI*::*cat* for allelic exchange in pKNG101, Cm^R, Sm^R	This study
pRX42	5′-protospacer-TG-3′ PAM *expI*-1 Δ*expI*::*cat* for allelic exchange in pKNG101, Cm^R, Sm^R	This study
pRX43	C6T *expI*-1 Δ*expI*::*cat* for allelic exchange in pKNG101, Cm^R, Sm^R	This study
Plasmids for *in vitro* transcription		
pGEM®-Teasy,	*In vitro* transcription cloning vector, T7 RNAP promoter Ap^R	Promega
pRP19	CRISPR2 spacer 2 and adjacent repeats in pGEM®-Teasy, Ap^R	This study
pRP20	CRISPR2 spacer 6 and adjacent repeats in pGEM®-Teasy, Ap^R	This study
pJSC6	Expression vector for N-terminal His Cas6f in pTRB30, Km^R	[5]
pTRB30	pQE-80L (Qiagen) based expression vector, Ap^R replaced by Km^R, Km^R	[5]

References

1. Guzman LM, Belin D, Carson MJ, Beckwith J (1995) Tight regulation, modulation, and high-level expression by vectors containing the arabinose PBAD promoter. J Bacteriol 177: 4121-4130.
2. Rose RE (1988) The nucleotide sequence of pACYC184. Nucleic Acids Res 16: 355.
3. Kaniga K, Delor I, Cornelis GR (1991) A wide-host-range suicide vector for improving reverse genetics in gram-negative bacteria: inactivation of the *blaA* gene of *Yersinia enterocolitica*. Gene 109: 137-141.
4. Grinter NJ (1983) A broad-host-range cloning vector transposable to various replicons. Gene 21: 133-143.
5. Przybilski R, Richter C, Gristwood T, Clulow JS, Vercoe RB, et al. (2011) Csy4 is responsible for CRISPR RNA processing in *Pectobacterium atrosepticum*. RNA Biol 8: 517-528.
