# Supplementary material for: Cytotoxic Chromosomal Targeting by CRISPR/Cas Systems Can Reshape Bacterial Genomes and Expel or Remodel Pathogenicity Islands
Source: PLoS Genet. 2013 Apr 18;9(4):e1003454. doi: 10.1371/journal.pgen.1003454 (PMC3630108; doi:10.1371/journal.pgen.1003454)
Supplement: Table S4 — Oligonucleotide primers used in this study. (PDF) [file pgen.1003454.s010.pdf]

Table S4. Oligonucleotide primers used in this study.

Name	Sequence (5'-3') (repeat nt in bold and point mutations bold and underlined)	Notes	Restriction site(s) (underlined)
Primers for sequencing inserts of CRISPR entry plasmids			
PF138	CACACTTTGCTATGCCATAG	F for pBAD30 MCS	
PF139	GCTACTGCCGCCAGG	R for pBAD30 MCS	
Primers for engineered CRISPR entry plasmids			
AM05	TTTCCCGGGAAAGGTAAAGCGCGATTCAC	F for pC1-780 plasmid	XmaI
TGO9	TTTCCCGGGACAGTTGCATGTGAAAGATG	F for pC1-180 plasmid	XmaI
RVO1	TTTCCCGGGAAAATGGCAATTGCTGC	F for pC1-52 plasmid	XmaI
RVO2	TTTCCCGGGAGTGATCGGGCTACAGTTC	F for pC1-16 plasmid	XmaI
TGO10	TTTGTCGACGAAGACTTTCTAAGCTGCCTGTACGG	R for pC1- plasmids	BbsI, SalI
Primers for non-specific spacers			
TGO12	TTTGAAGAC**CTTAGAAA**ACATCACACGAATCTTATGC	F for scrambled spacer 1	BbsI
TGO13	TTTGAAGAC**TTTCTAAGCTGCCTGTACGGCAGTGAAC**CGACCATCACAAGCATAAG	R for scrambled spacer 1	BbsI
TGO14	TTTGAAGAC**CTTAGAAA**CAGTACACGGTATGGATGC	F for scrambled spacer 2	BbsI
TGO15	TTTGAAGACTTTCTAAGCTGCCTGTACGGCAGTGAACCAGTTCAGCATCAGCATC	R for scrambled spacer 2	BbsI
TGO16	TTTGAAGAC**CTTAGAAA**ACTAAGTCCACTGAAACGT	F for scrambled spacer 3	BbsI
TGO17	TTTGAAGAC**TTTCTAAGCTGCCTGTACGGCAGTGAAC**ATTACGCCTTCAGACGTTTC	R for scrambled spacer 3	BbsI
Primers for *lacZ* spacers			
TGO54	TTTGAAGAC**CTTAGAAA**GTTACGTTGGTCTGACGGA	F for *lacZ* spacer 1	BbsI
TGO55	TTTGAAGAC**TTTCTAAGCTGCCTGTACGGCAGTGAAC**CTTCCAGATAACTTCCGTC	R for *lacZ* spacer 1	BbsI
TGO56	TTTGAAGAC**CTTAGAAA**CTGGATAACGACATCGGTA	F for *lacZ* spacer 2	BbsI
TGO57	TTTGAAGACTTTCTAAGCTGCCTGTACGGCAGTGAACTCGACTTCGCTGATAGGC	R for *lacZ* spacer 2	BbsI
PF279	TTTGAAGAC**CTTAGAAA**TACAGCGAACGCGTATCA	F for *lacZ* spacer 3	BbsI
PF280	TTTGAAGACTTTCTAAGCTGCCTGTACGGCAGTGAACCGCTGCACCATCCTTG	R for *lacZ* spacer 3	BbsI
Primers for *expI* spacers			
PF273	TTTGAAGAC**CTTAGAAA**TTGTTGTCAGAAACGAAATCAG	F for *expI* spacer 1	BbsI
PF274	TTTGAAGAC**TTTCTAAGCTGCCTGTACGGCAGTGAAC**AATAGCTCTTCTGATTTCGTTTC	R for *expI* spacer 1	BbsI
PF275	TTTGAAGAC**CTTAGAAA**TCAGAAAAGAGACGTTTAAAGATC	F for *expI* spacer 2	BbsI
PF276	TTTGAAGAC**TTTCTAAGCTGCCTGTACGGCAGTGAAC**ATTCAGTCGATCTTTAAACGTC	R for *expI* spacer 2	BbsI
PF277	TTTGAAGAC**CTTAGAAA**TATGCTAACGATATTAAAACGTTC	F for *expI* spacer 3	BbsI
PF278	TTTGAAGAC**TTTCTAAGCTGCCTGTACGGCAGTGAAC**CCCAGCCAGAACGTTTTAAT	R for *expI* spacer 3	BbsI
Primers for *eca0560* spacers			
PF326	TTTGAAGAC**CTTAGAAA**TCCAGTACTCAGGATCGTG	F for CRISPR2 spacer 6	BbsI
PF327	TTTGAAGAC**TTTCTAAGCTGCCTGTACGGCAGTGAAC**TTTATCGTACCAACACGATC	R for CRISPR2 spacer 6	BbsI
RV15	TTTGAAGAC**CTTAGAAA**TTCAGGTCAGTTATGTGAC	F for *eca0560* spacer 1	BbsI
RV14	TTTGAAGACTTTCTAAGCTGCCTGTACGGCAGTGAACCGAACCCACCGGCC	R for *eca0560* spacer 1	BbsI
Primers for creating *expI* mutant and chromosomal protospacer variants			
PF314	TTTGGTACCGGATCCGTGGCAATGATTACTCCATC	F for 5' 500bp of *expI*	KpnI, BamHI
PF315	CAAAACTCGAGAAAAGCTAGCTTCGCCTTCAGATTATTGTC	R for 5' 500bp of *expI*	XhoI, NheI
PF316	GCTAGCTTTTCTCGAGTTTTGTCGACTTTTAAGCTTCTATTGCACAGGCTTGATG	F for 3' 500bp of *expI*	NheI, XhoI, SalI, HindIII
PF317	TTTTCTAGACTGATGAATGGGTGAATCTC	R for 3' 500bp of *expI*	XbaI
PF322	ATCAGGGACGCTGGTTTG	F for mutant verification	
PF323	GCAGGGTAATCAAAGAGCAC	R for mutant verification	
TGO74	TTTGTCGACATACCGGGAAGCCCTGGG	F for *cat* resistance gene	SalI
TGO75	TTTAAGCTTAGGCGTTTAAGGGCACCA	R for *cat* resistance gene	HindIII
RV19	TTTCTCGAGTCCTTGTTGTCAGAAACGAAATCAGAAGAGCTATTATACCGGGAAGCCCTG	F for 5′-protospacer-GG-3′ PAM *expI* 1 protospacer	XhoI
RV20	TTTCTCGAGTC***A***TTGTTGTCAGAAACG	F for 5′-protospacer-TG-3′ PAM *expI* 1 protospacer	XhoI
RV21	TTTCTCGAGTCCTTGTT***A***TCAGAAACG	F for C6T *expI* 1 protospacer	XhoI
RV29	TTTCTCGAGTCCTT***A***TTGTCAGAAACG	F for C3T *expI* 1 protospacer	XhoI

Primers for CRISPR1 repeat mutants

RV22	CCCGGGAGTGATCGGGCTACA**GTTCACTGCCGTACAGGCAACTTAGAAA**TTG	F *expI* 1 repeat mutation G20A	XmaI
RV25	TTTGAAGAC**TTTCTAAGTTGCCTGTACGGCAGTGAAC**AATAGCTCTTCTGATTTCGTTTC	R *expI* 1 repeat mutation G20A	BbsI
RV23	CCCGGGAGTGATCGGGCTACA**GTTCACTGCCGTACAGGAAGCTTAGAAA**TTG	F *expI* 1 repeat mutation C18A	XmaI
RV26	TTTGAAGAC**TTTCTAAGCTTCCTGTACGGCAGTGAAC**AATAGCTCTTCTGATTTCGTTTC	R *expI* 1 repeat mutation C18A	BbsI
RV24	CCCGGGAGTGATCGGGCTACA**GTTCACTTCCGTACAGGAAGCTTAGAAA**TTG	F *expI* 1 repeat mutations C18A/G8U	XmaI
RV27	TTTGAAGAC**TTTCTAAGCTTCCTGTACGGCAGTGAAC**AATAGCTCTTCTGATTTCGTTTC	R *expI* 1 repeat mutation C18A/G8U	BbsI

Primers for checking HAI2 island loss/deletions (in Figure 6)

1 (TGO34)	AGGTGGATCCATGGATAACGCCTTTAGCC	F for *cas1* control	BamHI
2 (TGO35)	AGGTCTGCAGCAGAATGTTCATCGCACTAC	R for *cas1* control	PstI
3 (JCO8)	AGGTGGATCCATGAGCGATCGTTATGTCA	F for *eca0560*	BamHI
4 (JCO9)	AGGTGTCGACCTACTCACCTCCCACTTGC	R for *eca0560*	SalI
5 (attP_For)	TACGATGAAGCGAGAGCACA	F for *attP*/*attR*	
6 (attB-Rev)	ACGTAGCTCAAGCCAGTCGT	R for *attB*/*attR*	
7 (attB_For)	GATTCGTGGGGTGATTAAGG	F for *attB*/*attL*	
8 (attP_Rev)	CCGCCCCTTTGTCGAAATTA	R for *attP*/*attL*	

Additional primers for mapping HAI2 island loss/deletions (Figures S4 and S5)

Eca0516_F	CAGTCCACCGTCTTGAGTAT	F for *eca0516*
Eca0516_R	TCTTTCCAATGCGGGAATAG	R for *eca0516*
Eca0521_F	CGTTTAGAAAGCGGCACCTA	F for *eca0521*
Eca0521_R	CATTCATCATGCTGGCTGAG	R for *eca0521*
Eca0522_F	TACATACGCCGCTGTGAGTC	F for *eca0522*
Eca0522_R	GCGATAACGCACGATCAATA	R for *eca0522*
PilL_fwd	GTGCAACGACCCCTGTATCT	F for *eca0532*
PilL_rev_	TAGAGCGGCGTATCAACCTT	R for *eca0532*
PilP_fwd	CAGCGTTGCATCAGAAG	F for *eca0536*
PilP_rev	GACTGGTGAACATCCATCTTC	R for *eca0536*
PilU_fwd	GTATTGTACTGAAATGACGCTG	F for *eca0543*
PilU_rev	CATAAGGCCACCTCTATCAG	R for *eca0543*
TraE_fwd	CGTGGAGTCGTAGTGAATG	F for *eca0548*
TraE_rev	CTAACCCACTAGCCAATCTTG	R for *eca0548*
Eca0555_F	CATTGCAGTAAGGAACCG	F for *eca0555*
Eca0555_R	CATCAGAATGCCGCAAC	R for *eca0555*
JCP12	TTCTTGTACTGGTCGCGTTC	R for *eca0573*
JCP23	CATGGCAACATCAGCAGTAC	R for *eca0576*
JCP18	GGGACTCTATGACATGCTGAC	F for *eca0578*
JCP19	AGTCAGTGGCAACCTTACAC	R for *eca0578*
JCP16	GAATATTACAGTTGAAAAATCG	F for *eca0583*
JCP17	CCCTTAAGATCCTGATATCCC	R for *eca0583*
JCP20	AGGGAGGTAGTATGCGTATTTC	F for *eca0588*
JCP21	GCAGTGATAAAGGATCAAGATG	R for *eca0588*
PF330	TTTGGATCCGCCGATATTCATTATCG	F for *eca0599*
PF331	TTTTCTAGATGAAATACCTTATGCGCAC	R for *eca0599*
Cfa6_For	CAACGGGCATAACCTCAACT	F for *eca0603*
Cfa6_Rev	GTCGATGATTGCGCTGAGTA	R for *eca0603*
JCP9	ATGGACTGGACTAATATTCCCC	F for *eca0610*
JCP10	GCTGCCAATCTACAATTACCC	R for *eca0610*
Eca0613_F	CGAATAATAAAGGCGCGGTA	F for *eca0613*
Eca0613_R	CCAGCCTCCTTCTGAAACTG	R for *eca0613*
Eca0614_F	TCCTCATGCAGCGAATAGAA	F for *eca0614*
Eca0614_R	CGAAGGGCATCGAGTACAGT	R for *eca0614*

Primers for checking chromosomal *lacZ* loss/deletions (Figure S6)

AM01	TTTGAATTCAGGAGTAAAGCCATCATGAGCGAC	F for *lacZ*	EcoRI
AM02	TTTGGATCCTTAGTGACGTTGAATAGCGACGC	R for *lacZ*	BamHI
Eca14865_F	TCCGCCGGTACAACGGGTGT	F for *eca1482*	
Eca14865_R	AGAGCGTTGTGGCTCGTCAGC	R for *eca1482*	
NRPSF	CAGGTGGCTGAAGTCGGCGG	F for *eca1487*	
NRPSR	GTCGATCCGCTCAGCCTCGC	R for *eca1487*	
Eca14889_F	CATAGGCCAGATGGGCGGCG	F for *eca1488*	
Eca14889_R	CGGTTGCCCTGCTCACCCAG	R for *eca1488*	

Primers for *in vitro* transcription vectors

RP44	CGTTAACCGCTCAGTGAC	F for CRISPR2 spacer 5
RP45	GACGTTTAACGAGTGTCAGAAC	R for CRISPR2 spacer 7
RP46	CTGAACCCTCCGATTATC	F for CRISPR2 spacer 1
RP47	CAATATGATGCTTAGTTACAATATCTGC	R for CRISPR2 spacer 3
